# Cancer-associated SPOP mutations enlarge nuclear size and facilitate nuclear envelope rupture upon farnesyltransferase inhibitor treatment

**DOI:** 10.1172/JCI189048

**Published:** 2025-07-15

**Authors:** Zixi Wang, Lei Li, Qi Ye, Yuzeshi Lei, Mingming Lu, Leihong Ye, Jialu Kang, Wenyue Huang, Shan Xu, Ke Wang, Jing Liu, Yang Gao, Chenji Wang, Jian Ma, Lei Li

**Affiliations:** 1 Department of Urology, The First Affiliated Hospital of Xi’an Jiaotong University, Xi’an, China.; 2 State Key Laboratory of Genetic Engineering, MOE Engineering Research Center of Gene Technology, Shanghai Engineering Research Center of Industrial Microorganisms, School of Life Sciences, Fudan University, Shanghai, China.

**Keywords:** Cell biology, Therapeutics, Cancer, Drug therapy, Molecular biology

## Abstract

Nuclear size is crucial for cellular functions and often increases with malignancy. Irregular nuclei are linked to aggressive tumors, driven by genetic and epigenetic changes. However, the precise mechanisms controlling nuclear size are still not fully understood. In this study, we demonstrated that cancer-associated speckle-type POZ protein (SPOP) mutations enlarged nuclear size by reducing the protein level of lamin B2 (LMNB2), a key nuclear integrity protein. Mechanistically, SPOP bound to LMNB2 and promoted its mono-ubiquitination at lysine-484, which protected it from degradation by the E3 ubiquitin ligase WD repeat domain 26. SPOP mutations disrupted this process, leading to reduced LMNB2 levels and impaired nuclear envelope (NE) integrity. This compromised NE was more vulnerable to damage from farnesyltransferase inhibitors (FTIs), causing nuclear rupture in SPOP-mutant tumor cells. This study identified SPOP as a positive regulator of nuclear size; the findings suggest tumors with SPOP mutations may be vulnerable to FTI-based therapies.

## Abstract 2

Cancer-associated SPOP mutations increase nuclear size by reducing LMNB2, compromising nuclear envelope integrity and potentially sensitizing tumors to farnesyltransferase inhibitor therapies.

## Graphical abstract

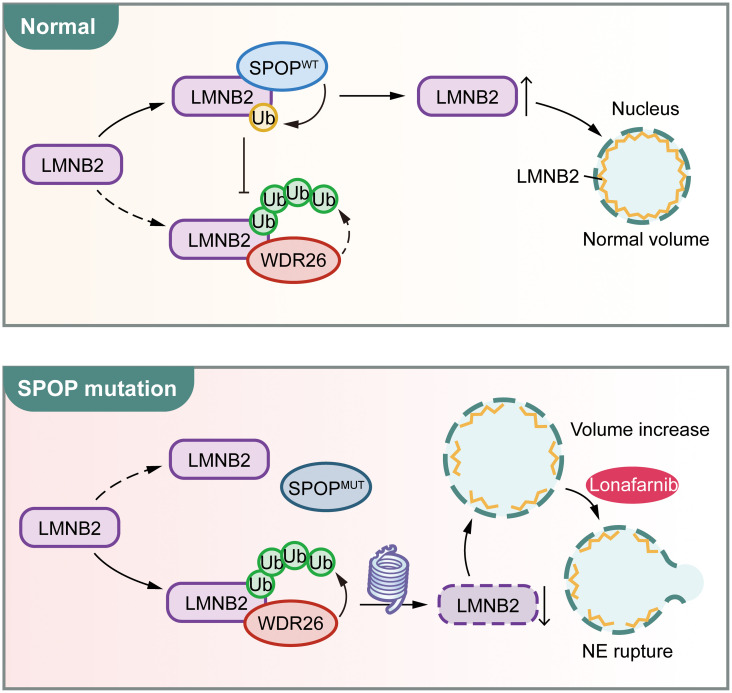

## Introduction

Nuclear size is a key feature of cellular architecture, reflecting the intricate balance of cellular processes that govern growth, division, and function. In healthy cells, nuclear size is tightly regulated to maintain proper gene expression, chromatin organization, and cellular homeostasis (1, 2). However, in cancer, this regulation often becomes disrupted, leading to alterations in nuclear size—a hallmark of malignancy (3). Enlarged and irregularly shaped nuclei are frequently observed in various aggressive cancers, including those of the breast, prostate, colon, and pancreas (2, 4–6). Regulation of nuclear size—via nuclear envelope (NE) integrity (7–9), nuclear lamins (10, 11), and nucleocytoplasmic transport (12–16)—is essential for cellular order. Disruptions in these processes can lead to NE rupture, chromosomal instability, and increased metastatic potential (17–19). Understanding this interplay could pave the way for new targeted therapies, improving treatment options for aggressive cancers.

Nuclear lamins play a central role in controlling nuclear size by forming the nuclear lamina, a dense fibrous network that lines the inner surface of the NE in eukaryotic cells (6, 10). This network provides essential mechanical support, maintaining the nucleus’s shape, integrity, and organization (20). Nuclear lamins are divided into A type and B type. A-type lamins, such as lamin A and lamin C, contribute to nuclear stability and chromatin organization (21–25), whereas B-type lamins, including lamin B1(LMNB1) and LMNB2, are key to NE formation and maintenance (4, 26, 27). LMNB1 is degraded through the lysosome (28), but the regulation of LMNB2 in cancer is less well understood (29).

The *SPOP* gene encodes a substrate-binding adaptor subunit of the CULLIN3 (CUL3)-RING box 1 E3 ubiquitin ligase complex (30–33). SPOP is linked to oncogenesis through frequent mutations in cancers such as prostate and endometrial cancers (34–37). It targets several cancer-related proteins for degradation, including androgen receptor (AR) (38–40), BRD4 (41), SRC3 (42), and ATF2 (43). Additionally, SPOP mediates nondegradative ubiquitination of substrates like geminin (44), SQSTM1 (45, 46), and 53BP1 (47). Our previous research showed that SPOP regulates protein recruitment in the ER (48). Given the continuity between nuclear and ER membranes, changes in ER morphology could influence nuclear size (9, 12, 49). However, it remains unclear whether SPOP plays a role in nuclear size control.

In the present study, we showed that cancer-associated SPOP mutations increased nuclear size by decreasing LMNB2 protein levels. Normally, SPOP bound to LMNB2 and facilitated its mono-ubiquitination, which prevented degradation by WDR26. However, SPOP mutations led to enhanced LMNB2 degradation and disrupted NE integrity, causing nuclear rupture and making cancer cells more sensitive to farnesyltransferase inhibitor–based (FTI-based) therapies.

## Results

### SPOP mutation increases cell nuclear volume.

**Figure 1:**
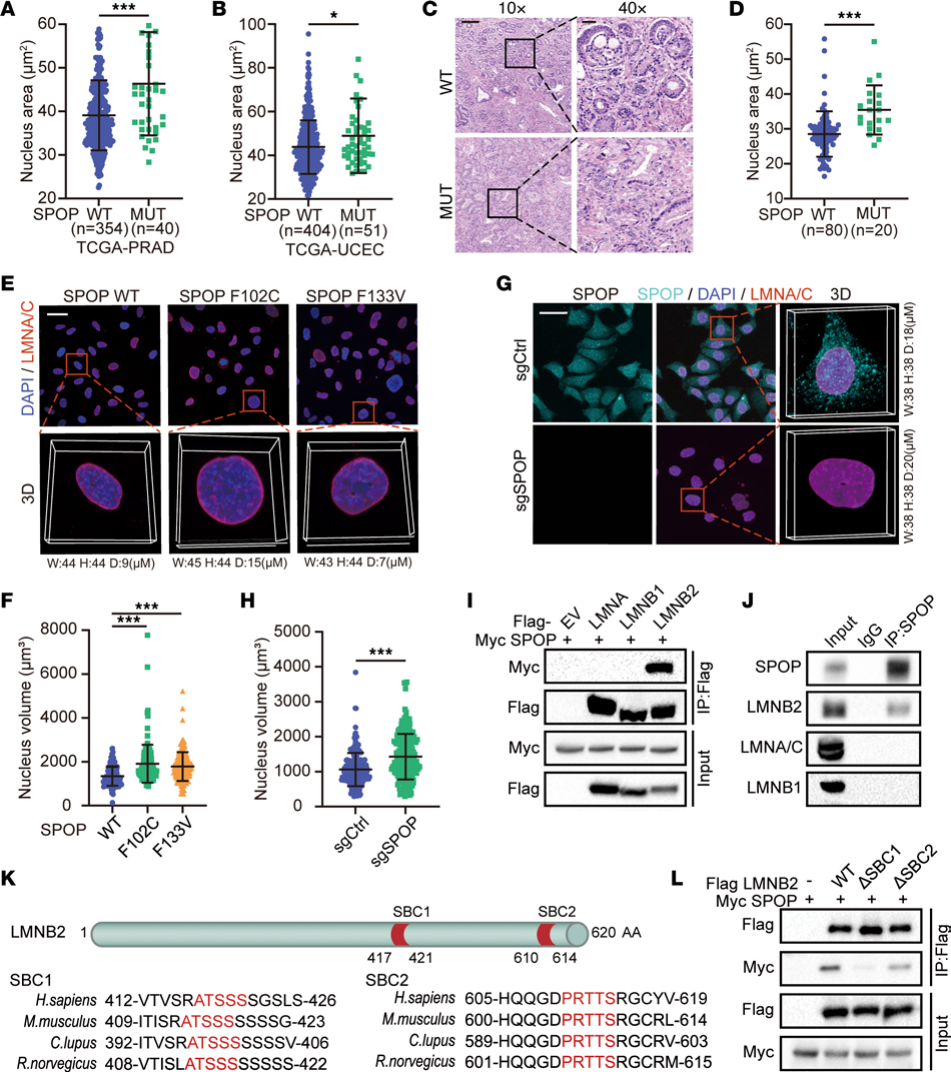
SPOP mutation increases cell nuclear volume. (**A** and **B**) Quantification of nuclear area from diagnostic slides from patients in the SPOP WT (*n* = 354) and SPOT mutant (MUT) (*n* = 40) groups from the TCGA prostate adenocarcinoma data set (37) (**A**) and patients in the SPOP WT (*n* = 404) and SPOP MUT (*n* = 51) groups from the TCGA uterine corpus endometrial carcinoma data set (50) (**B**). (**C** and **D**) Representative H&E staining (**C**) images of prostate cancer specimens from the SPOP WT (*n* = 80) and SPOP MUT (*n* = 20) groups and quantification data (**D**). Scale bars: 200 μm in ×10 fields; 40 μm in ×40 fields. (**E** and **F**) PC-3 cells infected with lentivirus expressing WT, F102C, or F133V SPOP were analyzed using 2D and 3D IF (**E**) and quantified (**F**). Scale bar: 50 μm. (**G** and **H**) Control or *SPOP* knockout PC-3 cells were analyzed using 2D and 3D IF (**G**) and quantification (**H**). Scale bar: 50 μm. Data are shown as the mean ± SD of 3 biological replicates (*n* > 200). (**I**) Co-IP analysis of indicated proteins in 293T cells transiently transfected with Flag-LMNA, LMNB1, or LMNB2. (**J**) Co-IP analysis of endogenous proteins in 293T cells using indicated antibodies. (**K**) A LMNB2 structure diagram showing 2 putative evolutionally conserved SBC motifs (SBC1 and SBC2) located at the C-terminal of LMNB2. (**L**) Co-IP analysis of indicated proteins in 293T cells transiently transfected with Flag-LMNB2 WT, ΔSBC1, or ΔSBC2. *P < 0.05, ***P < 0.001 by Mann-Whitney test (**A**, **B**, **D**, and **H**) or 1-way ANOVA followed by Dunnett’s multiple comparisons test (**F**).

Histopathology images from The Cancer Genome Atlas (TCGA) prostate cancer data set (37), uterine corpus endometrial cancer data set (50), and our cohort revealed that SPOP-mutant tumors had larger nuclei compared with SPOP WT tumors (Figure 1, A–D). To investigate this further, we examined the impact of 2 common SPOP mutations, F102C and F133V, on nuclear size. Stable expression of these mutants increased nuclear volume in PC-3 and HeLa cells compared with the SPOP WT group (Figure 1, E and F, and Supplemental Figure 1, A–D; supplemental material available online with this article; https://doi.org/10.1172/JCI189048DS1). Similar results were observed in *SPOP* knockout cells (Figure 1, G and H, and Supplemental Figure 1, E–H). Given the central role of lamin proteins in nuclear size control (21, 22, 27, 51), we explored whether lamin involvement was linked to the enlarged nuclei in SPOP-associated cases. Co-immunoprecipitation (co-IP) assays showed that both ectopically expressed and endogenous SPOP interacted with LMNB2, but not with lamin A/C (LMNA) or LMNB1(Figure 1, I and J, and Supplemental Figure 1, I–K). This interaction was further confirmed in vitro, where LMNB2 directly bound to GST-SPOP purified from *E*. *coli* (Supplemental Figure 1L). Proteins targeted by SPOP typically contain a SPOP-binding consensus sequence (SBC) (Φ-π-S-S/T-S/T where Φ is a nonpolar residue and π is a polar residue) (52). Notably, LMNB2 contains 2 SBC motifs (SBC1: ^417^ATSSS^421^; SBC2: ^610^PRTTS^614^), which are absent in LMNA and LMNB1. Despite the high sequence similarity among LMNA, LMNB1, and LMNB2 (53), these SBC sites are unique to LMNB2 and conserved across species (Figure 1K and Supplemental Figure 1M). We further demonstrated that deletion of the 5 amino acids or mutation of ^418^TSS^420^ to ^418^TAA^420^ in SBC1 completely abolished SPOP’s interaction with LMNB2 in HEK293T cells (Figure 1L and Supplemental Figure 1N), confirming ^417^ATSSS^421^ as a functional SBC motif in LMNB2. Collectively, our data showed that cancer-associated SPOP mutation increased nuclear volume, likely through direct binding with LMNB2.

### SPOP mutation destabilizes LMNB2 protein levels.

**Figure 2:**
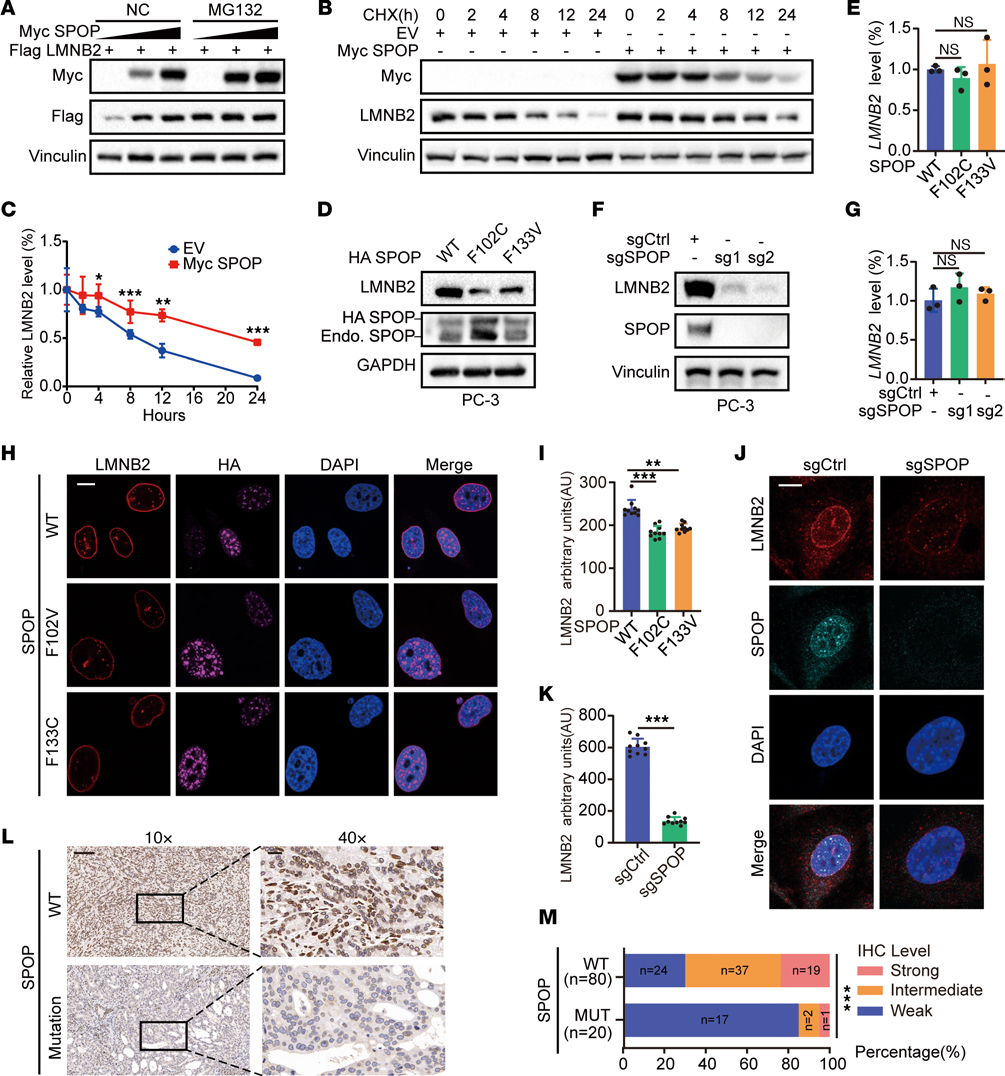
SPOP mutation impairs LMNB2 protein levels. (**A**) IB analysis of whole-cell lysates (WCLs) derived from 293T cells with indicated plasmids. DMSO or 10 μM MG132 was added for 12 hours before harvest. (**B** and **C**) IB analysis (**B**) and quantification (**C**) of LMNB2 protein in WCLs from 293T cells transfected with or without SPOP after cycloheximide treatment. Data are shown as the mean ± SD of 3 biological replicates (*n* = 3). (**D**–**G**) IB analysis of WCL derived from PC-3 cells infected with lentivirus expressing WT, F102C or F133V SPOP (**D**) and PC-3 control and *SPOP* knockout cells (**F**). The *LMNB2* RNA levels are shown in (**E**) and (**G**). Data are shown as the mean ± SD of 3 independent experiments (*n* = 3). (**H** and **I**) PC-3 cells infected with lentivirus expressing HA-WT, F102C, or F133V SPOP were subjected to IF. Representative images are shown in (**H**) and quantification in (**I**). Scale bar: 10 μm. Data are reported as the mean ± SD of 10 fields (>200 cells; *n* = 10) from 3 biological replicates. (**J** and **K**) PC-3 control and *SPOP* knockout cells were subjected to IF; representative images are shown in (**J**) and quantification in (**K**). Scale bar: 10 μm. Data are reported as the mean ± SD of 10 fields (>200 cells; *n* = 10) of 3 biological replicates. (**L** and **M**) Representative images of IHC staining (**L**) of LMNB2 antibodies on prostate cancer specimens from SPOP WT (*n* = 80) and SPOP MUT (*n* = 20) groups. The distribution of LMNB2 IHC levels is shown in (**M**). Scale bar: 200 μm in ×10 fields; 40 μm in ×40 fields. **P < 0.01 and ***P < 0.001 by 2-way ANOVA followed by Tukey’s multiple comparisons test (**C**) or 1-way ANOVA followed by Dunnett’s multiple comparisons test (**E**, **G**, and **I**) or 2-tailed unpaired Student’s *t* test (**K**) or Fisher’s exact test (**M**).

Given the canonical role of SPOP as an E3 ubiquitin ligase, we investigated whether SPOP affects LMNB2 protein levels. Surprisingly, increased SPOP concentrations led to elevated LMNB2 protein levels (Figure 2A) and extended the half-life of endogenous LMNB2 in PC-3 cells (Figure 2, B and C). Consistently, SPOP mutations or knockout reduced LMNB2 protein levels in PC-3 and HeLa cells without affecting *LMNB2* mRNA levels (Figure 2, D–G, and Supplemental Figure 2, A–D). The proteasome inhibitor MG132 treatment rescued LMNB2 protein level in both SPOP-mutant or knockout PC-3 and HeLa cells (Supplemental Figure 2, E and F). Immunofluorescence (IF) assays confirmed that SPOP mutation or knockout decreased LMNB2 signals compared with controls (Figure 2, H–K, Supplemental Figure 2, G and H, and Supplemental Figure 3, A and B). Moreover, LMNB2 overexpression successfully rescued the increased nuclear size induced by SPOP mutations and knockout in both PC-3 and HeLa cells (Supplemental Figure 3, C–J).

To further assess the impact of SPOP mutations on LMNB2 protein levels in patient specimens, we analyzed 100 primary prostate tumors from our cohort (Supplemental Table 1). Sanger sequencing identified 20 tumors with SPOP mutations. IHC revealed that 85% of SPOP-mutated tumors showed weak LMNB2 staining. In contrast, only 30% of SPOP-WT tumors had weak LMNB2 staining, with the majority (70%) exhibiting strong or intermediate staining (Figure 2, L and M). These findings suggested that LMNB2 protein levels were impaired in SPOP-mutated prostate cancer specimens.

### SPOP maintains LMNB2 protein level by promoting its mono-ubiquitination at lysine-484.

**Figure 3:**
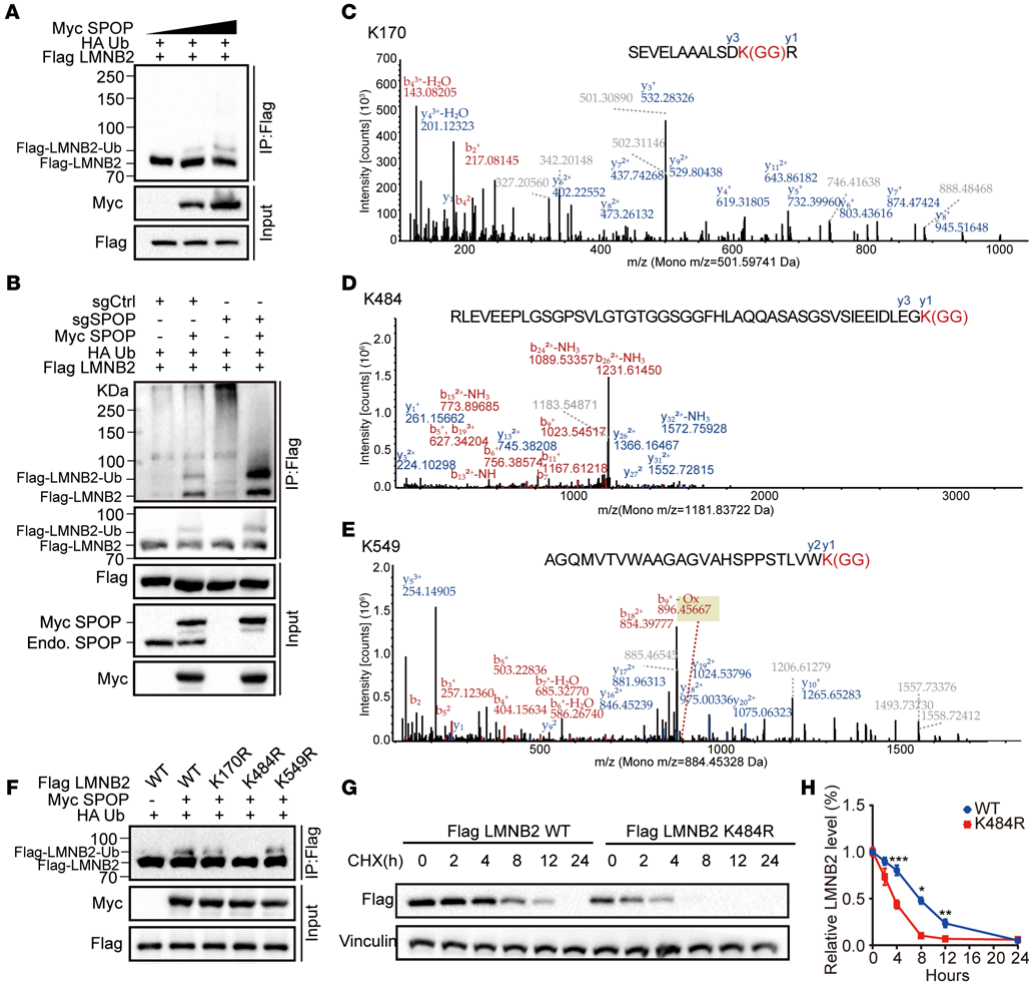
SPOP maintains LMNB2 protein level by promoting its mono-ubiquitination at lysine-484. (**A**) Co-IP analysis of indicated proteins in 293T cells transfected with increased Myc-SPOP WT in combination with Flag-LMNB2 and HA-Ub. (**B**) Co-IP analysis of indicated proteins in control or *SPOP* knockout 293T cells transfected with the indicated plasmids. (**C**–**E**) Mass spectrometry analysis revealed LMNB2 ubiquitination at lysine residues 170(**C**), 484 (**D**), and 549 (**E**). (**F**) Co-IP analysis of indicated proteins in 293T cells transfected with Flag-WT or mutated LMNB2 in combination with other constructs. (**G** and **H**) IB analysis (**G**) and quantification (**H**) of Flag-LMNB2 protein in whole-cell lysates from 293T cells transfected with Flag-WT or K484R LMNB2 after cycloheximide treatment. Data are reported as the mean ± SD of 3 biological replicates (*n* = 3). (**H**) Statistical comparisons were performed using 2-way ANOVA followed by Tukey’s multiple comparisons test. *P < 0.05, **P < 0.01, and ***P < 0.001.

To further investigate how SPOP influences LMNB2 protein level, we examined whether LMNB2 is a ubiquitinating substrate of SPOP. Intriguingly, although SPOP knockout reduced total LMNB2 half-life (Supplemental Figure 4, A and B), we found that increased expression of SPOP did not induce the smeared pattern typical of polyubiquitination of LMNB2 but instead led to a prominent increase in a discreet band consistent with mono-ubiquitin addition (Figure 3A). Knockout of endogenous SPOP by CRISPR-Cas9 greatly attenuated both exogenous and endogenous LMNB2 mono-ubiquitination in HEK293T cells, and this effect was reversed by restored expression of SPOP (Figure 3B and Supplemental Figure 4C). Moreover, deletion of the SBC1 region of LMNB2 also inhibited its mono-ubiquitination, indicating that SPOP binding was necessary for LMNB2 mono-ubiquitination (Supplemental Figure 4D). To determine which lysine residues of LMNB2 are ubiquitinated by SPOP, we performed mass spectrometry on HEK293T cells transfected with Flag-tagged LMNB2, Myc-tagged SPOP, and HA-tagged ubiquitin plasmids. Ubiquitination at lysine residues 170, 484, and 549 in LMNB2 was detected by mass spectrometry (Figure 3, C–E). Mutagenesis analysis showed that mutation of Lys484 abolished SPOP-dependent LMNB2 mono-ubiquitination and reduced the half-life of LMNB2 in HEK293T cells (Figure 3, F–H). These findings indicated that SPOP maintained LMNB2 protein levels by promoting its mono-ubiquitination at Lys484.

### Lys484 mono-ubiquitination stabilizes LMNB2 by antagonizing WDR26-mediated degradation.

**Figure 4:**
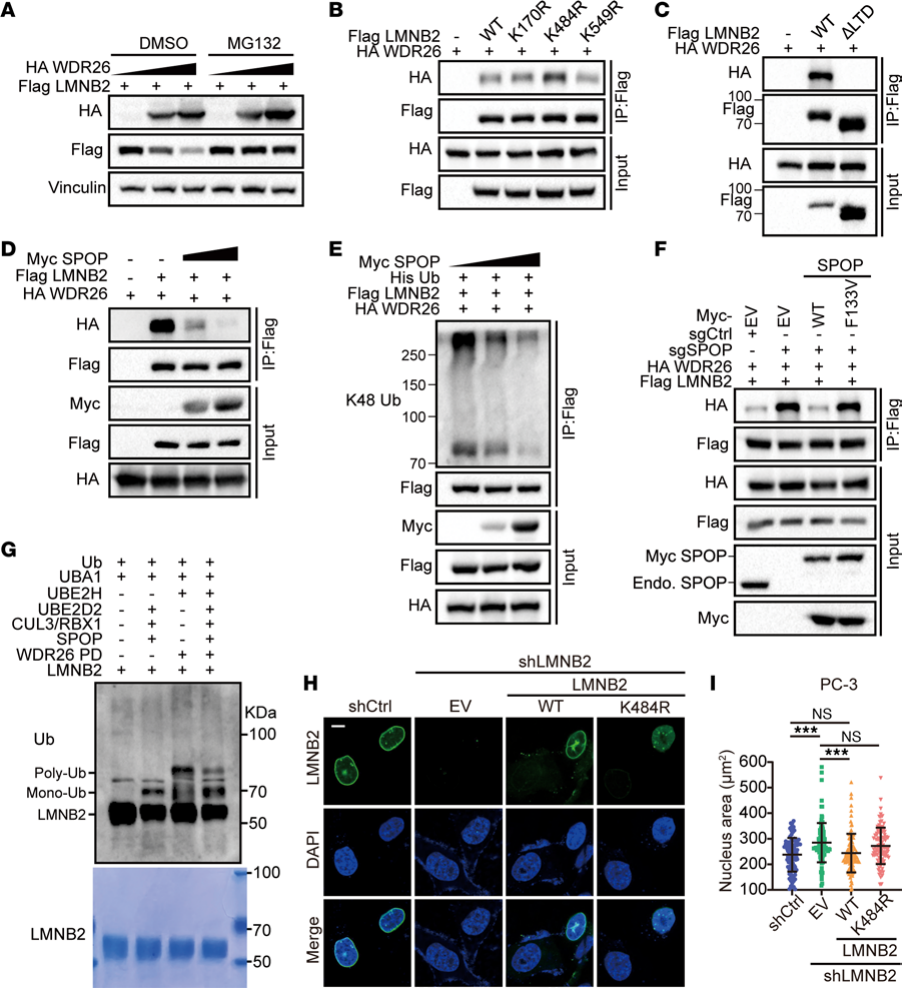
Lys484 mono-ubiquitination stabilizes LMNB2 by antagonizing WDR26-mediated degradation. (**A**) IB analysis of whole-cell lysates (WCLs) derived from 293T cells with indicated plasmids. DMSO or 10 μM MG132 was added for 12 hours before harvest. (**B**) Co-IP analysis of indicated proteins in 293T cells transfected with Flag-WT or mutated LMNB2 in combination with other constructs. (**C**) Co-IP analysis of indicated proteins in 293T cells transiently transfected with Flag-LMNB2 WT or ΔLTD. (**D** and **E**) Co-IP analysis of indicated proteins (**D**) and K48 ubiquitination (**E**) in 293T cells transfected with increased Myc SPOP in combination with indicated constructs. (**F**) Co-IP analysis of indicated proteins in 293T control and *SPOP* knockout cells transfected with indicated plasmids. (**G**) Ubiquitination of LMNB2 using *E*. *coli*–purified proteins in vitro. (**H** and **I**) PC-3 control and *LMNB2* knockdown cells transfected with Flag-WT or K484R LMNB2 were subjected to IF; representative images are shown in (**H**) and quantification in (**I**). Scale bar: 10 μm. Data are reported as the mean ± SD of 3 biological replicates (*n* = 100). (**I**) Statistical comparisons were performed using 1-way ANOVA followed by Dunnett’s multiple comparisons test. ***P < 0.001.

Our finding that SPOP promoted mono-ubiquitination and prevented LMNB2 degradation (Figure 3) led us to hypothesize that SPOP stabilized LMNB2 by counteracting K48-linked, ubiquitination-dependent degradation mediated by another E3 ligase. Previous studies have identified WDR26 as a core subunit of the GID ubiquitin ligase complex that regulates the polyubiquitination and degradation of LMNB2 in zebrafish and mouse erythroblasts (29). We confirmed that WDR26-mediated LMNB2 degradation was blocked by the proteasome inhibitor MG132 in HEK293T cells (Figure 4A), suggesting that WDR26 facilitated proteasomal degradation of LMNB2. Notably, although the WT LMNB2 bound to WDR26, the K484R mutation, but not other mutations, enhanced both the exogenous and endogenous WDR26 binding (Figure 4B and Supplemental Figure 5A). Deletion of the Lamin-tail (LTD) domain, which includes Lys484, abolished LMNB2-WDR26 binding, indicating that mono-ubiquitination of LMNB2 at Lys484 blocked its interaction with WDR26 (Figure 4C). Indeed, increased SPOP expression reduced LMNB2 binding to WDR26 and decreased K48-linked polyubiquitination of LMNB2 (Figure 4, D and E). Conversely, *SPOP* knockout increased LMNB2-WDR26 binding and K48-linked polyubiquitination, which was reversed by the expression of WT SPOP but not the F133V mutant (Figure 4F and Supplemental Figure 5, B and C). We conducted in vitro ubiquitination assays using *E*. *coli*–purified proteins and confirmed that SPOP mediates LMNB2 mono-ubiquitination. Notably, although WDR26 facilitated LMNB2 polyubiquitination, the presence of SPOP-driven mono-ubiquitination inhibited this process (Figure 4G). Consistently, the K484R mutant LMNB2 failed to rescue the nuclear size increase caused by *LMNB2* knockdown (Figure 4, H and I, and Supplemental Figure 5, D–G). These findings suggested that SPOP-mediated mono-ubiquitination of LMNB2 at Lys484 inhibited WDR26 binding and degradation of LMNB2, thereby stabilizing LMNB2 protein levels and maintaining nuclear size.

### SPOP mutation increases NE rupture risk upon farnesyltransferase inhibition.

**Figure 5:**
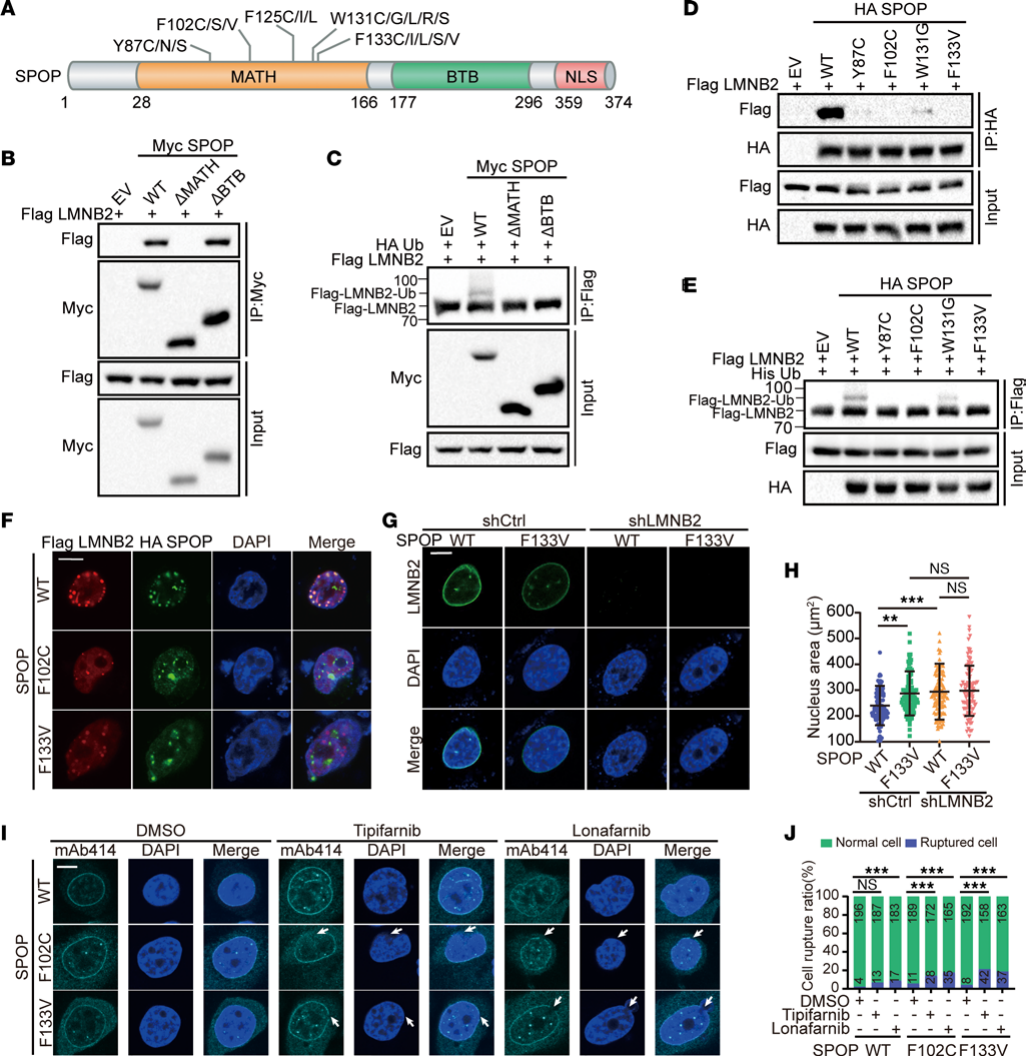
SPOP mutation increases NE rupture risk upon farnesyltransferase inhibition. (**A**) Schematic of domain organization of SPOP and major SPOP mutations in prostate cancers. (**B** and **C**) Co-IP analysis of indicated proteins (**B**) and ubiquitination (**C**) in 293T cells transfected with Myc-WT, ΔMATH, or ΔBTB SPOP in combination with other constructs. (**D** and **E**) Co-IP analysis of indicated proteins (**D**) and ubiquitination (**E**) in 293T cells transfected with Myc-WT or mutant SPOP in combination with other constructs. (**F**) Representative images of IF of Flag LMNB2 and HA SPOP from PC-3 cells. Scale bar: 10 μm. (**G** and **H**) PC-3 control and *LMNB2* knockdown cells infected with lentivirus expressing WT or F133V SPOP were subjected to LMNB2 IF; representative images are shown in (**G**) and quantification in (**H**). Scale bar: 10 μm. Data are reported as the mean ± SD of 3 biological replicates (*n* = 100). (**I** and **J**) PC-3 cells infected with lentivirus expressing HA-WT, F102C, or F133V SPOP were subjected to mAb414 IF. DMSO, 10 μM tipifarnib, or 5 μM lonafarnib was added for 24 hours before harvest. Representative images are shown in (**I**), quantification of the cell rupture ratio is shown in (**J**). Scale bar: 5 μm. Data are reported as the mean ± SD of 3 biological replicates (*n* = 200). ***P < 0.001 by 1-way ANOVA followed by Dunnett’s multiple comparisons test (**H**) or Fisher’s exact test (**J**).

SPOP mutations in prostate cancer predominantly occur within the MATH domain, which is essential for substrate binding (37, 54) (Figure 5A). Using co-IP assays, we found that the SPOP ΔMATH mutant loses its ability to bind and monoubiquitinate LMNB2. In contrast, the ΔBTB mutant, which lacks CUL3 binding and is unable to ubiquitinate substrates, retained its binding ability to LMNB2 but failed to promote its mono-ubiquitination (Figure 5, B and C, and Supplemental Figure 6A). We further generated 5 prostate cancer–associated SPOP mutants, and co-IP assays revealed that all 5 mutants showed impaired binding to LMNB2 compared with WT SPOP (Figure 5D). These mutations also reduced SPOP-mediated mono-ubiquitination of LMNB2 (Figure 5E). IF assays in PC3 and HeLa cell lines visually confirmed the colocalization of LMNB2 with WT SPOP, but not with the mutant forms (Figure 5F and Supplemental Figure 6B). Moreover, whereas *LMNB2* knockdown resulted in an increase in nuclear size, the expression of either WT or mutant SPOP showed no significant effect on nuclear size control in both PC-3 and HeLa cells (Figure 5, G and H, and Supplemental Figure 6, C–F). These results indicated that pathophysiological mutations in SPOP compromised its ability to monoubiquitinate LMNB2 and regulate nuclear size.

Given that LMNB2 is crucial for maintaining NE integrity, we investigated the effects of SPOP mutations on the NE. We observed that the NE rupture rate was slightly higher in SPOP-mutant cells compared with SPOP WT cells. FTIs such as tipifarnib and lonafarnib are known to disrupt normal lamin maturation and cause abnormal lamin localization, which can weaken NE stability (25, 55, 56). To determine whether farnesyltransferase inhibition further affects NE rupture in SPOP-mutant cells, we treated these cells with the FTIs. Although FTI treatment did not affect NE rupture in SPOP WT cells compared with the control, SPOP-mutant cells exhibited a substantial increase in NE rupture (Figure 5, I and J, and Supplemental Figure 6, G and H). These findings suggested that SPOP-mutant cells were more vulnerable to NE rupture when farnesyltransferase was inhibited.

### SPOP-mutant cells are hypersensitive to farnesyltransferase inhibition.

**Figure 6:**
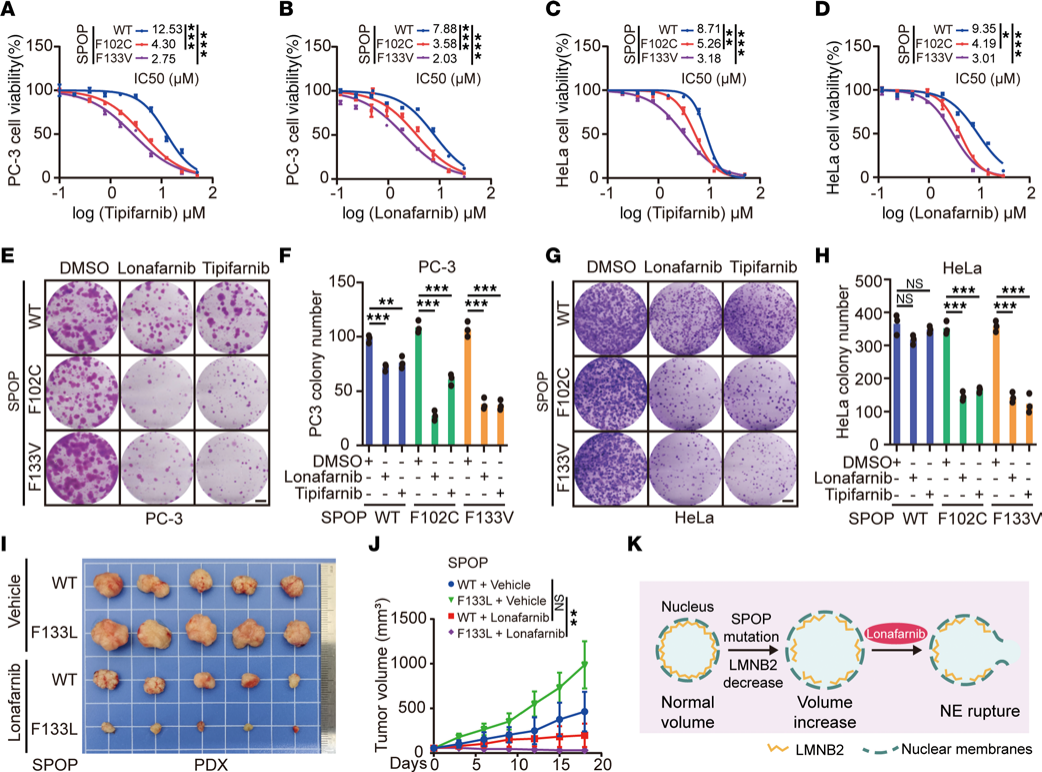
SPOP mutant cells are hypersensitive to farnesyltransferase inhibition. (**A**–**D**) Dose-response survival curves of PC-3 (**A** and **B**) and HeLa (**C** and **D**) cell lines infected with lentivirus expressing HA-WT, F102C, or F133V SPOP exposed to increasing concentrations of tipifarnib (**A** and **C**) or lonafarnib (**B** and **D**). Data are reported as the mean ± SD of 3 independent experiments (*n* = 3). (**E**–**H**) Colony-formation assays in PC-3 (**E** and **F**) and HeLa (**G** and **H**) cell lines infected with lentivirus expressing HA-WT, F102C, or F133V SPOP. The number of colonies was counted. Representative colonies are shown in (**E** and **G**); quantification data are shown in (**F** and **H**). Data are presented as the mean ± SD of 3 independent experiments (*n* = 3). (**I** and **J**) SPOP WT or SPOP F133L PDX tumors were transplanted subcutaneously into SCID mice and treated with lonafarnib (20 mg/kg, twice daily by oral gavage) or vehicle. Mice were treated for 18 days and then sacrificed. Tumors were isolated and are shown in (**I**) and their volumes (*n* = 5) are shown in (**J**). All data are reported as mean ± SD. (**K**) Schematic illustrating the mechanism of FTIs effectively killing SPOP-mutant cells. *P < 0.05, **P < 0.01, and ***P < 0.001 by 2-way ANOVA (**A**–**D**) or 1-way ANOVA followed by Dunnett’s multiple comparisons test (**F** and **H**) or 2-way ANOVA followed by Tukey’s multiple comparisons test (**J**).

Because SPOP-mutant cells showed an increase in NE rupture upon farnesyltransferase inhibition (Figure 5, I and J, and Supplemental Figure 6, G and H), we hypothesized that these cells are hypersensitive to FTIs due to NE rupture. To test this, we evaluated the viability of SPOP mutant–expressing PC-3, C4-2, and HeLa cells treated with 2 FTIs, tipifarnib, and lonafarnib. A dose-response survival assay showed that the SPOP-mutant F133V resulted in a lower IC_50_ for both inhibitors compared with the empty vector control groups in PC-3, C4-2, and HeLa cells (Figure 6, A–D, and Supplemental Figure 7, A and B). FTI treatment also inhibited the growth of SPOP-mutant cells, leading to fewer and smaller colonies, whereas control cells had only a slight reduction in colony size and number (Figure 6, E–H, and Supplemental Figure 7, C and D). Similar to the in vitro results, the addition of lonafarnib markedly reduced SPOP F133V PC-3 tumor growth in vivo compared with the WT xenograft (Supplemental Figure 7, E and F). We further examined the effect of FTI treatment using lonafarnib in a clinically relevant setting with a patient-derived xenograft (PDX) model from a prostate cancer metastatic lesion harboring the SPOP F133L mutation and AR expression (Supplemental Figure 7G). Consistently, SPOP F133L mutant PDX tumors were more sensitive to lonafarnib compared with SPOP-WT PDX tumors (Figure 6, I and J). These results demonstrated that SPOP-mutated prostate cancer cells were hypersensitive to FTIs both in vitro and in vivo (Figure 6K).

## Discussion

The size of the nucleus directly influences the tension on the NE. Larger nuclei, resulting from dysregulated nuclear size control, increase the surface area and tension on the NE, stretching the nuclear lamina and potentially causing damage, especially if there are mutations or deficiencies in lamina components like LMNA/C (25, 57–59). This weakened lamina structure compromises NE integrity, leading to more frequent and severe ruptures (25, 55, 56). Loss of NE integrity has been associated with normal aging and various human diseases, including cancer (13, 60). For instance, in Hutchinson-Gilford progeria syndrome, the abnormal accumulation of immature LMNA leads to nuclear morphology changes, such as NE lobulation and thickening of the lamina (22). In cancer, key steps of tumor cell invasion require deformation of the NE to navigate through the 3D tissue environment (61–63). Although NE rupture can promote cancer progression, it may also expose a vulnerability in metastatic cancer cells that could be targeted by antimetastatic drugs, such as FTIs (25, 55, 56). In 2020, the FDA approved lonafarnib for treating Hutchinson-Gilford progeria syndrome and other laminopathies (64), and tipifarnib has progressed to phase 3 clinical trials for advanced pancreatic cancer and acute myeloid leukemia (65, 66). Our findings showed that SPOP-mutant cancer cells were at a higher risk of NE rupture and may be more susceptible to farnesyltransferase inhibition, providing a potential therapeutic avenue for targeting cancers with a high risk of NE rupture, such as those with SPOP mutations.

Ubiquitination is a key post-translational modification in which ubiquitin is covalently attached to substrate proteins (67, 68). This modification can occur as mono-ubiquitination, in which a single ubiquitin molecule is added to a protein, or as polyubiquitination, where ubiquitin chains are formed on the substrate (69). These 2 types of ubiquitination have distinct cellular roles, with mono-ubiquitination often regulating nondegradative processes like DNA repair and receptor trafficking (70, 71), whereas polyubiquitination usually targets proteins for degradation by the proteasome (72). The type of ubiquitination is determined by specific ubiquitin ligases (E3s), which confer substrate specificity, and deubiquitinating enzymes, which remove ubiquitin moieties to reverse the signal. The balance between these enzymes allows dynamic regulation of ubiquitination, enabling cells to adapt to various internal and external stimuli (67, 72). Whereas SPOP-mediated polyubiquitination of multiple substrates is well documented (47, 73), no monoubiquitinated substrates of SPOP have been reported until now. In this study, we demonstrated that SPOP can promote mono-ubiquitination of LMNB2 at lysine 484, which inhibited WDR26-mediated polyubiquitination of LMNB2. This competition between mono- and polyubiquitination stabilized LMNB2, protecting it from proteasomal degradation and extending the traditional understanding that mono-ubiquitination primarily regulates nonproteolytic processes.

In conclusion, our findings demonstrated that SPOP played a critical role in regulating nuclear size by promoting the mono-ubiquitination of LMNB2 at lysine 484, thereby preventing WDR26-mediated degradation. We also showed that cancer-associated mutations in SPOP led to decreased levels of LMNB2 and compromised NE integrity, making SPOP-mutant cells more susceptible to NE rupture and vulnerable to farnesyltransferase inhibition.

## Methods

### Sex as a biological variable.

Our study exclusively examined male mice because the disease modeled prostate cancer is only relevant in males.

### Cell lines.

Human HeLa (catalog CCL-2; ATCC) and HEK293T (catalog CRL-11268; ATCC) cells were cultured in DMEM (high glucose; catalog 11965092; Gibco) containing 10% FBS) (catalog 30067334; Thermo Fisher Scientific) and 1% penicillin-streptomycin (catalog G4003; Servicebio). PC-3 (catalog CRL-1435; ATCC) and C4-2 (catalog CRL-3314; ATCC) cells were cultured in RPMI 1640 medium (catalog 21870092; Gibco) with 10% FBS and 1% penicillin-streptomycin. All cells were maintained in a humidified incubator at 37°C with 5% CO_2_. Routine mycoplasma testing was performed and all cells tested negative.

### Antibodies.

Primary antibodies used were SPOP (catalog 16750-1-AP; Proteintech Group; 1:1,000 [antibody dilution]), lamin A/C (catalog A0249; ABclonal; 1:1,000), lamin B1 (catalog A16909; ABclonal; 1:1,000), rabbit monoclonal LMNB2 (catalog ab151735; Abcam; 1:1,000), mouse monoclonal LMNB2 (catalog ab8983; Abcam; 1:1,000), Flag (catalog 8146; Cell Signaling Technology; 1:1,000), HA (catalog 3724; Cell Signaling Technology; 1:1,000). Flag-tag pAb–HRP–DirecT (catalog PM020-7; MBL Life Science; 1:1,000), GAPDH (catalog A19056; ABclonal; 1:1,000), K48-linkage specific polyubiquitin (catalog 8081; Cell Signaling Technology; 1:1,000), ubiquitin (catalog 43124; Cell Signaling Technology; 1:1,000), nuclear pore complex (catalog ab24609; Abcam; 1:200), HA-tag-Alexa Fluor 647 (catalog M180-A64; MBL Life Sciences; 1:200). Myc (catalog sc-40; Santa Cruz Biotechnology; 1:500), vinculin (catalog sc-73614; Santa Cruz Biotechnology; 1:1,000), IgG (H + L), FITC (catalog EK023; Zhuangzhi Biotechnology; 1:200), rabbit IgG (H + L), Cy3 (catalog EK022; Zhuangzhi Biotechnology; 1:200), mouse IgG (H + L), FITC (catalog EK013; Zhuangzhi Biotechnology; 1:200), mouse IgG (H + L) and Cy3 (catalog EK012; Zhuangzhi Biotechnology; 1:200).

### Plasmids.

Constructs for SPOP pCMV3-SPOP-Myc, pCMV3-SPOP F102C-Myc, pCMV3-SPOP F133V -Myc, pCMV3-SPOP ΔMATH-Myc, pCMV3-SPOP ΔBTB-Myc, pCMV3-SPOP ΔBACK-Myc were derived in-house pCMV-LMNA (human)-3×FLAG-Neo (P5464), pCMV-LMNB1(human)-3×FLAG-Neo (catalog P43765), pEnCMV-LMNB2-3×FLAG and pCMV-WDR26-3×HA-Neo (catalog P54648) were bought from Miaoling Biology. pCMV3-SPOP-HA, pCMV3-SPOP Y87C-HA, pCMV3-SPOP F102C-HA, pCMV3-SPOP W131G-HA, pCMV3-SPOP F133V-HA, pTsin-CMV-SPOP-HA, pTsin-CMV-SPOP F102C-HA and pTsin-CMV-SPOP F133V-HA, pGEX-4T-1-LMNB2, pEnCMV-LMNB2 ΔSBC1-3×FLAG, pEnCMV-LMNB2 ΔSBC2-3×FLAG, pEnCMV-LMNB2 K170R-3×FLAG, pEnCMV-LMNB2 K484R-3×FLAG, pEnCMV-LMNB2 K484R-3×FLAG, pEnCMV-LMNB2 K549R-3×FLAG, and pEnCMV-LMNB2 ΔLTD-3×FLAG were constructed in this study. Primer sequences are listed in Supplemental Table 1.

### Animal models.

Male nude mice and NOD/SCID mice, 5 weeks old, were obtained from the Laboratory Animal Center of Xi’an Jiaotong University.

### Detection of prostate cancer specimens with SPOP mutations by Sanger sequencing.

Prostate cancer tissue samples were obtained from the First Affiliated Hospital of Xi’an Jiaotong University (Xi’an, China), with approval from the hospital’s ethical committee. Informed consent was obtained from all patients.

For Sanger sequencing, DNA was extracted tissue specimens from all 100 patients with prostate cancer, using a QIAamp DNA FFPE Tissue kit (catalog 56404; QIAGEN). PCR was performed using 2 × Hot Start Taq Master Mix (catalog E028-02A; Novoprotein), and PCR products were purified using a GeneJET Extraction kit (catalog K0692; Fermentas) according to the manufacturer’s instructions and used for Sanger sequencing. The primers of Amp-Exon6 and Amp-Exon7 used for DNA amplification were listed in Supplemental Table 1. Amp-Exon6-Reverse and Amp-Exon7-Forward were also used for Sanger sequencing. The pathology numbers of the 100 tissue samples and the corresponding SPOP-mutation status are listed in Supplemental Table 2.

### H&E and IHC staining.

FFPE tumor tissue samples were sectioned into thin slices with a thickness of 4 μm. The slides were stained with H&E solutions or LMNB2 antibodies following standard H&E and IHC protocols. Images were captured using a PANNORAMIC Midi II (3DHISTECH) and analyzed with CaseViewer 2.3. (3DHISTECH) For IHC-stained slides, protein expression quantification was conducted using established scoring criteria. The proportion of stained cells (%) and staining intensity (0 = no staining; 1 = weak staining; 2 = intermediate staining; 3 = strong staining) were evaluated, and these values were multiplied to yield a score ranging from 0 to 3, using ImageJ (National Institutes of Health) for analysis.

### Cell transfection and virus infection.

Cells were transfected with the indicated plasmids using either polyethylenimine (catalog 23966-2; Polysciences) or Lipofectamine 2000 (catalog 11668019; Thermo Fisher Scientific), following the manufacturers’ instructions. After transfection, cells were cultured for 48 hours before harvesting for further experiments. For lentiviral infection, cells were used to package pLKO shRNAs or pLenti-CRISPRV2 GFP plasmids. To generate stable cell lines, cells were incubated with the viral supernatant in the presence of 2 mg/mL polybrene. Infected cells were selected with puromycin (1 mg/mL) for a minimum of 3 days. The sgRNA sequences for *SPOP* knockout and shRNA sequences for *LMNB2* knockdown were provided in Supplemental Table 1.

### IF assay.

PC-3 or HeLa cells were seeded onto 13 mm glass coverslips. After washing once with PBS, the coverslips were fixed in 4% formaldehyde (catalog BC1016; ZHHC) for 20 minutes at room temperature. Prior to incubation with primary antibodies, the coverslips were permeabilized in 0.2% Triton X-100 in PBS for 15 minutes. After 3 additional washes with PBS, the samples were incubated with PBS containing 5% bovine serum albumin and 5% glycerol for 1 hour at room temperature. Afterward, the samples were incubated with primary antibodies overnight and then subjected to at least 3 washes with PBS. Subsequently, the samples were incubated with secondary fluorescence–conjugated antibodies for 1 hour at room temperature in the dark. Finally, after 3 washes with PBS, the coverslips were stained using Antifade Mounting Medium with DAPI (catalog AP0271S; Accuref Scientific), then mounted on glass slides and visualized using an Olympus FV3000 Laser Scanning Confocal Microscope.

### Cell nucleus size quantification.

Diagnostic slides and gene mutation data of patients with prostate cancer or uterine corpus endometrial cancer reported in the TCGA were respectively downloaded from the Genomic Data Commons (https://gdc.cancer.gov) and the cBioPortal for Cancer Genomics (https://www.cbioportal.org/) databases. Cell nucleus size was quantified using the ImageJ software. For nucleus area, more than 1,000 cells from randomly selected figures of each H&E-stained slide and more than 100 cells in 10 randomly picked photographs from IF were analyzed. For nucleus volume, 10 randomly picked 3D photographs of PC-3 and Hela cells that included more than 150 cells were used to calculated after 3D reconstruction.

### Mean LMNB2 AU quantification.

LMNB2 relative fluorescence intensity from IF images was quantified using ImageJ software. For each analyzed cell line, 10 randomly selected images containing more than 100 cells were used to standardize the integration of optical density, measured in AU.

### IBs and co-IP.

Cells were collected and lysed using an IP buffer (50 mM Tris–HCl, pH 7.4, 150 mM NaCl, 0.1% NP-40) containing protease inhibitor (catalog 04693132001; Sigma-Aldrich) and phosphatase inhibitor (catalog 04906837001; Sigma-Aldrich). The lysate was centrifuged at 13,000*g* for 15 minutes at 4°C, and the supernatant was harvested. Protein concentration was measured using Pierce BCA Protein Assay (catalog YH375034&YH372327; Thermo Fisher Scientific) and an Epoch Microplate Spectrophotometer (BioTek) at 562 nm. Proteins were prepared by mixing with 5× SDS loading buffer (250 mM Tris–HCl, pH 6.8, 10% SDS, 25 mM β-mercaptoethanol, 30% glycerol, and 0.05% bromophenol blue) and boiled for 10 minutes.

Equal amounts of proteins were loaded onto an SDS-PAGE gel for electrophoresis, followed by transfer onto a nitrocellulose membrane (catalog 75936355; Pall Corp.). The membrane was blocked with 5% milk for 1 hour at room temperature, then incubated with the primary antibody overnight at 4°C. The next day, the membrane was washed 3 times with 1× TBST (20 mM Tris, 100 mM NaCl, 0.1% Tween-20) for 10 minutes and incubated with secondary antibodies or anti-DDDDK-tag -HRP antibody for 1 hour at room temperature. Protein bands were detected using ECL Western blotting substrate (Bio-Rad) and visualized with Image Lab (Bio-Rad).

For co-IP analysis, cells were lysed using the same IP buffer on ice for at least 10 minutes. After centrifuging at 16,000*g* for 15 minutes at 4°C, the supernatant was incubated with either primary antibody–conjugated protein A/G beads (catalog 20423; Thermo Fisher Scientific) or HA (catalog A2095; Sigma Aldrich)/Flag (catalog A2220; Sigma Aldrich)/Myc (catalog A7470; Sigma Aldrich) conjugated agarose beads while rotating overnight at 4°C. The following day, beads were washed at least 4 times with IP buffer on ice. After being mixed with 1.5× SDS loading buffer and boiled for 10 minutes, the proteins were analyzed using IB analysis.

### In vivo ubiquitination assay.

HEK293T cells were transfected with HA-/His-tagged ubiquitin along with the indicated plasmids. At 48 hours after transfection, the cells were treated with 20 μM MG132 (catalog 133407-826; Sigma Aldrich) for 6 hours to inhibit proteasomal degradation. Cells were then lysed in IP buffer supplemented with protease and phosphatase inhibitors and incubated on ice for longer than 10 minutes. The lysate was sonicated and centrifuged at 16,000*g* for 15 minutes at 4°C. The supernatant was incubated with Flag-conjugated agarose beads while rotating overnight at 4°C. The following day, the beads were washed 4 times with IP buffer on ice. The bound proteins were eluted and subjected to IB analysis.

### In vitro ubiquitination assays.

The commercial E2 Select Ubiquitin Conjugation Kit (catalog 20440ES10; YEASEN) was used for in vitro ubiquitination assays. *E*. *coli*–purified LMNB2, SPOP, or co-IP-pulldown WDR26 was incubated with the ubiquitination reaction mix including 100 mM Ubiquitin, 100 nM human UBE1, 1 mM human UBE2H or 1 mM human UBE2D, and 1 mM Mg-ATP at 37°C for 12 hours. After the reaction, samples were mixed with 1× SDS loading buffer, boiled for 10 minutes, and then analyzed using IB.

### Protein half-life assays.

Cells were treated with cycloheximide (catalog 2112S; Cell Signaling Technology; 100 mg/mL) for the indicated time before harvesting, and protein abundances were measured by IB analysis. The protein abundance was quantified using the “Analyze Gels” function in ImageJ software, and the endogenous LMNB2 or Flag-LMNB2 bands were normalized to vinculin, then normalized to the 0 time point.

### Real-time qPCR.

Total RNA was extracted from cells using RNAfast 200 reagents (catalog 220010; Fastagen) following the manufacturer’s instructions. RNA concentration was measured using an Epoch Microplate Spectrophotometer at 260 nm. Reverse transcription was performed using PrimeScript Real-Time Master Mix (catalog RRO36A; TAKARA). Relative mRNA levels were quantified via real-time qPCR using 2× SYBR Green qPCR Master Mix (catalog TSE202; Tsingke), with gene expression normalized to 18S rRNA levels. The comparative Ct method was applied to assess the relative expression of the indicated genes. The required primer sequences are provided in Supplemental Table 1.

### LMNB2 ubiquitination site analysis by mass spectrometry.

HEK293T cells were transfected with Flag-LMNB2, HA-Ub, and Myc-SPOP plasmids. After 48 hours, the proteins were extracted using IP buffer supplemented with protease and phosphatase inhibitors. The lysates were incubated on ice for 10 minutes, followed by centrifugation at 16,000*g* for 15 minutes at 4°C. Subsequently, 100 μL of Flag-conjugated agarose beads were added to the supernatant and incubated overnight at 4°C. The beads were washed at least 4 times with IP buffer, and proteins were eluted by adding 1.5× SDS loading buffer and heating at 100°C for 10 minutes. The supernatant was then collected and subjected to downstream liquid chromatography–mass spectrometry analysis, a process technically supported by Novogene. Protein identification and quantification were performed using Proteome Discoverer 2.5 (Thermo Fisher Scientific). Ubiquitination sites were identified as a mass shift of 114 Da on lysine residues, which corresponds to the di-glycine (GG) remnant left after tryptic digestion of ubiquitinated proteins. The identified ubiquitinated peptides also were manually inspected to confirm the correct peptide sequences and modification sites.

### Cell viability assays.

A total of 3,000 cells per well were plated in 96-well plates and cultured in 200 μL of the indicated medium containing 10% serum. After 24 hours, the medium was replaced with fresh medium, and cells were treated with various concentrations of compounds in 200 μL of medium for 72 hours. Cell viability was assessed using the MTT assay (catalog 298-93-1; Sigma Aldrich) assay, following the manufacturer’s instructions. Absorbance was measured at 490 nm using an Epoch microplate spectrophotometer. All experiments were performed in triplicate.

### Colony-formation assays.

An appropriate number of cells were seeded in 6-well plates in the indicated medium. After 24 hours, cells were treated with DMSO or the specified lonafarnib (catalog HY-15136; MedChemExpress) or tipifarnib (HY-10502;, MedChemExpress) doses and cultured for 1 to 2 weeks (the culture medium was replaced with fresh medium every 3 days), depending on colony size. Cells were then fixed with 4% paraformaldehyde for 15 minutes and stained with 0.5% (w/v) crystal violet for 30 minutes. After gentle washing with running water, the plates were allowed to dry, and the number of colonies in each group was counted and analyzed.

### Drug treatment of PDX and xenograft tumors.

All mice were housed under standard pathogen-free conditions with a 12-hour light/dark cycle and had ad libitum access to food and water. PDX tumors, including SPOP WT and F133L mutants, were expanded by passaging tumor pieces (~1 mm³) subcutaneously into 6- to 8-week-old NOD/SCID male mice. PC-3 cells (5 × 10^–6^) infected with lentivirus expressing pTsin-SPOP WT or pTsin-SPOP F133V mutant were injected subcutaneously into 6- to 8-week-old nude mice to form xenografts tumors. Once tumors reached approximately 100 mm³ (~3 weeks after transplantation), tumor-positive animals were randomly divided into different treatment groups (*n* = 5 mice/group). Mice were treated with vehicle control or lonafarnib (20 mg/kg, twice daily by oral gavage) for 18 consecutive days. Tumor growth was measured using calipers every 3 to 4 days. Tumor volume was calculated using the formula: 0.5 × length × width². Upon completion of measurements, graft tumors were harvested for photography.

### Statistics.

Graphs were generated using Prism 10 (GraphPad Software). All numerical data are presented as mean ± SD or specific values, as required. Differences between groups were analyzed using *t* tests, Fisher’s exact test, or 2-way ANOVA, using Prism 10 for statistical computing. *P* values of less than 0.05 were considered significant.

### Study approval.

All animal procedures were conducted in accordance with the guidelines approved by the Institutional Animal Care and Use Committee of Xi’an Jiaotong University.

### Data availability.

All data needed to evaluate the conclusions in this article are present in the article and/or the supplemental materials. Values for all data points shown in graphs are reported in the Supporting Data Values. The ubiquitination mass spectrometry raw data are available via iProX partner repository (74) (accession: IPX0009798000; https://www.iprox.cn/). Additional data related to this study may be requested from the authors.

## Author contributions

JM designed the experiments. JM, ZW, and Lei Li (second listed author) designed and performed most experiments, analyzed the data. QY performed the detection of prostate cancer specimens with SPOP mutations by Sanger sequencing. YL generated the SPOP-KO cells. ML, LY, and JK performed the subcutaneous implantation of PDX tumors. LY, WH, SX, and KW provided administrative and technical support. Lei Li (last listed author) and JM acquired funding. JM, ZW, and Lei Li (second listed author) prepared the manuscript. Lei Li (last listed author), JM, and CW supervised the study. Lei Li (last listed author), JM, CW, JL, and YG finalized the manuscript. ZW and Lei Li (second listed author) are co–first authors and contributed equally to this work; authorship order reflects that the study was initiated by ZW, who was joined by Lei Li (second listed author) in leading the project.

## Supplementary Material

Supplemental data

Unedited blot and gel images

Supporting data values

## Version 1 07/15/2025

Electronic publication
