# Supplementary material for: Cancer-associated SPOP mutations enlarge nuclear size and facilitate nuclear envelope rupture upon farnesyltransferase inhibitor treatment
Source: J Clin Invest. 2025 Jul 15;135(14):e189048. doi: 10.1172/JCI189048 (PMC12259254; doi:10.1172/JCI189048)
Supplement: Supplemental data [file jci-135-189048-s118.pdf]

Supplemental material

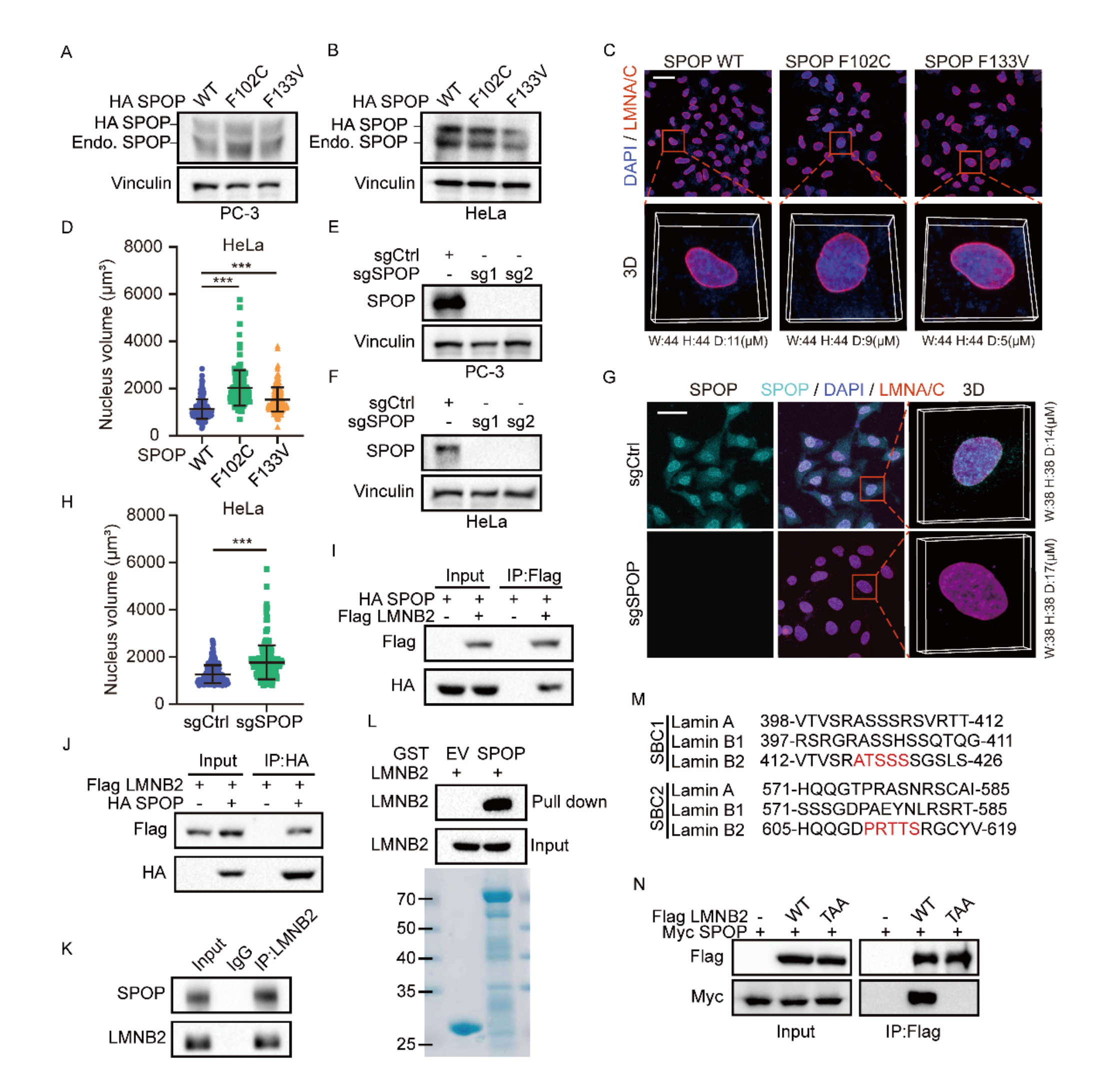

Supplemental Figure 1. SPOP mutation increases cell nuclear volume, Related to Figure 1. (**A** and **B**) IB analysis of whole cell lysates (WCL) derived from PC-3 cells (**A**) and HeLa cells (**B**) infected with lentivirus expressing WT, F102C or F133V SPOP. (**C** and **D**) HeLa cells infected with lentivirus expressing WT, F102C or F133V SPOP were analyzed using 2D and 3D IF (**C**) and quantified in (**D**). Scale bar, 50μm. Data were shown as the mean ± SD of three biological replicates ($n > 150$). (**E** and **F**) IB analysis of WCL derived from *Control* or *SPOP* knockout PC-3 cells (**E**) and HeLa cells (**F**). (**G** and **H**) *Control* or *SPOP* knockout HeLa cells were analyzed using 2D and 3D IF (**G**) and quantification in (**H**). Scale bar, 50μm. Data were shown as the mean ± SD of three biological replicates ($n > 200$). (**I** and **J**) Co-IP analysis of indicated proteins in 293T cells transiently transfected with Flag-LMNB2 and HA-SPOP. (**K**) Co-IP analysis of endogenous proteins in 293T cells using the indicated antibodies. (**L**) Co-IP of LMNB2 protein *in vitro* pulled down by GST or GST-SPOP recombinant proteins purified from *E. coli*. (**M**) Amino acid alignment of

SBC motifs in LMNB2 compared to LMNA and LMNB1. (**N**) Co-IP analysis of indicated proteins in 293T cells transiently transfected with Flag-LMNB2 WT or TAA mutants. ****P* < 0.001 by One-way ANOVA followed by Dunnett's multiple comparisons test (**D**) or Mann-Whitney test(**H**).

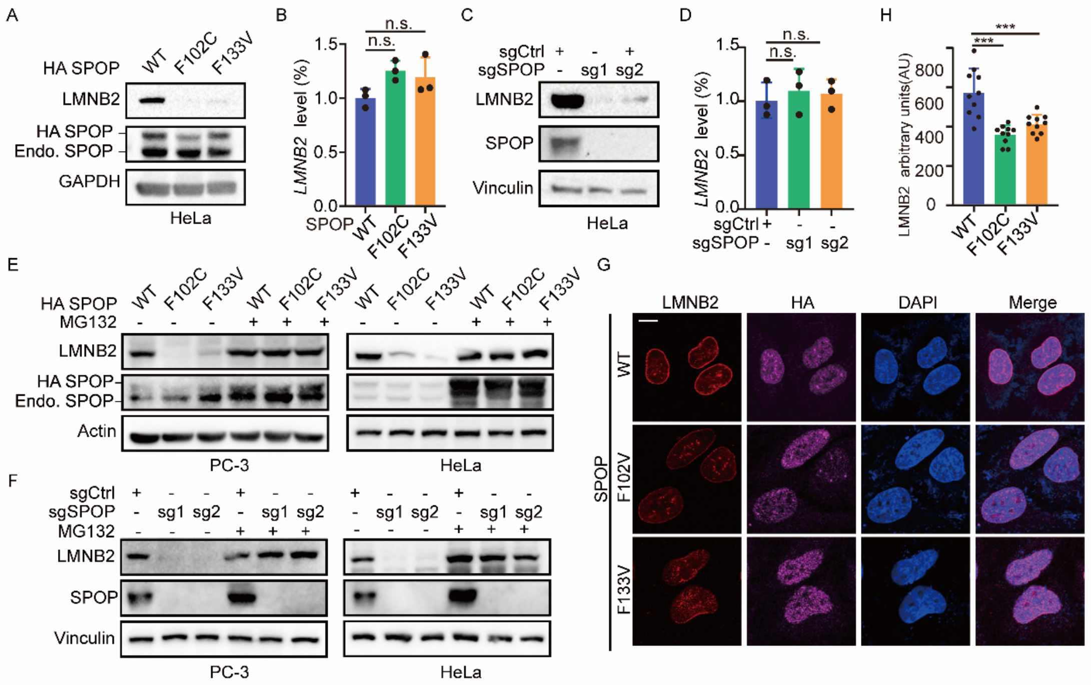

Supplemental Figure 2. SPOP mutation impairs LMNB2 protein levels, Related to Figure 2.

(**A**-**D**) IB analysis of WCL derived from HeLa cells infected with lentivirus expressing WT, F102C or F133V SPOP (**A**) and HeLa *control* and *SPOP* knockout cells (**C**). The *LMNB2* RNA levels were shown in (**B**) and (**D**). Data were shown as the mean ± SD from three independent experiments ($n = 3$). (**E** and **F**) IB analysis of WCL derived from PC-3 (**E**) and HeLa (**F**) cells with indicated conditions. (**G** and **H**) HeLa cells infected with lentivirus expressing HA-WT, F102C or F133V SPOP were subjected to IF with representative images shown in (**G**) and quantification in (**H**). Scale bar, 10μm. Data were shown as the mean ± SD of 10 fields (>200 cells, $n = 10$) from three biological replicates. (**B**, **D** and **H**) Statistical comparisons were performed by One-way ANOVA followed by Dunnett's multiple comparisons test. n.s., not significant, ***$P < 0.001$.

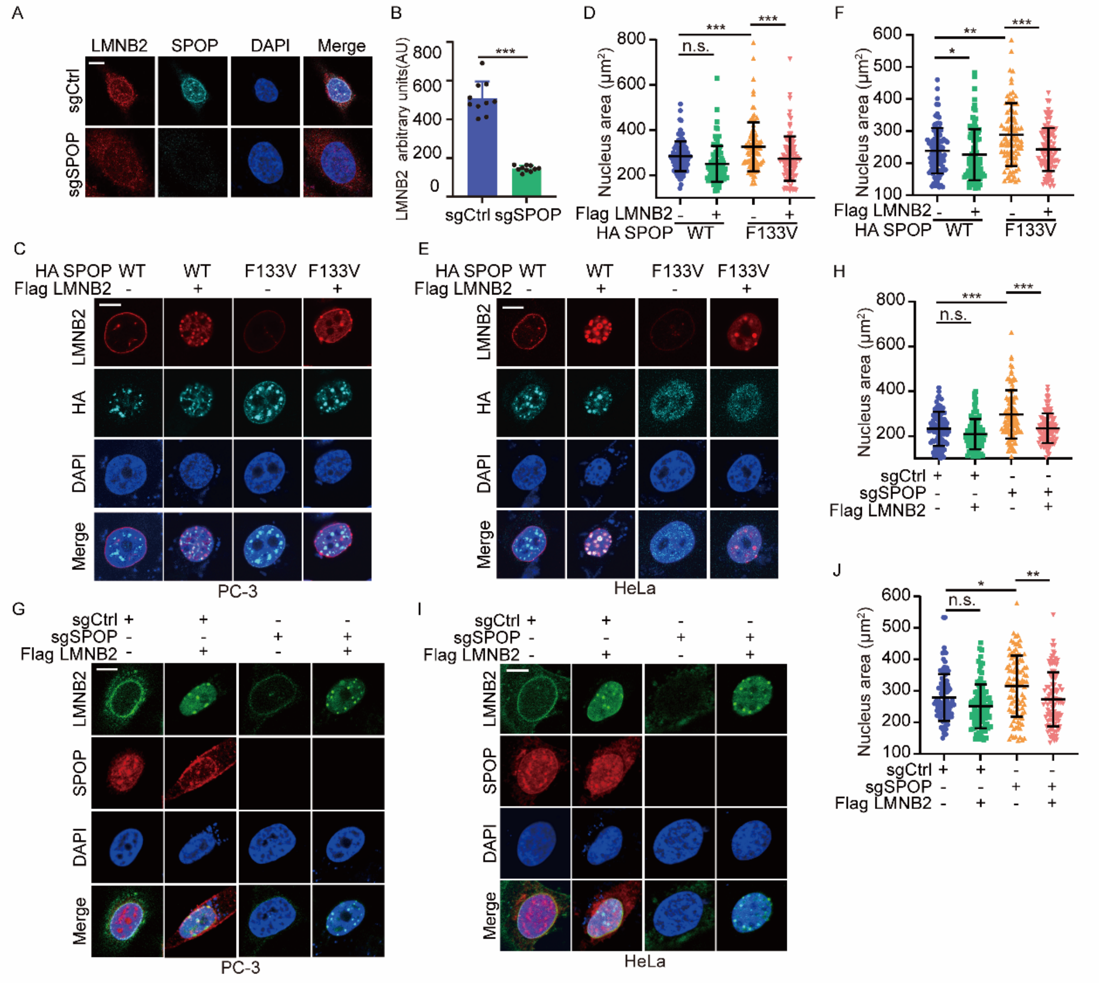

Supplemental Figure 3. SPOP mutation destabilizes LMNB2 protein, Related to Figure 2.

(**A** and **B**) HeLa *control* and *SPOP* knockout cells were subjected to IF with representative images shown in (**A**) and quantification in (**B**). Scale bar, 10μm. Data were shown as the mean ± SD of 10 fields (>200 cells, *n* = 10) from three biological replicates. (**C-F**) PC-3 (**C** and **D**) and HeLa (**E** and **F**) cells infected with lentivirus expressing HA-WT, F102C or F133V SPOP were subjected to IF with representative images shown in (**C** and **E**) and quantification in (**D** and **F**). Scale bar, 10μm. Data were shown as the mean ± SD of three biological replicates (*n* = 100). (**G-J**) PC-3 (**G** and **H**) and HeLa (**I** and **J**) HeLa *control* or *SPOP* knockout cells were subjected to IF with representative images shown in (**G** and **I**) and quantification in (**H** and **J**). Scale bar, 10μm. Data were shown as the mean ± SD of three biological replicates (*n* = 100). n.s., not significant, **P* < 0.05, ***P* < 0.01 and ****P* < 0.001 by Two-tailed unpaired Student's *t*-test (**B**) or One-way ANOVA followed by Dunnett's multiple comparisons test (**D**, **F**, **H** and **J**).

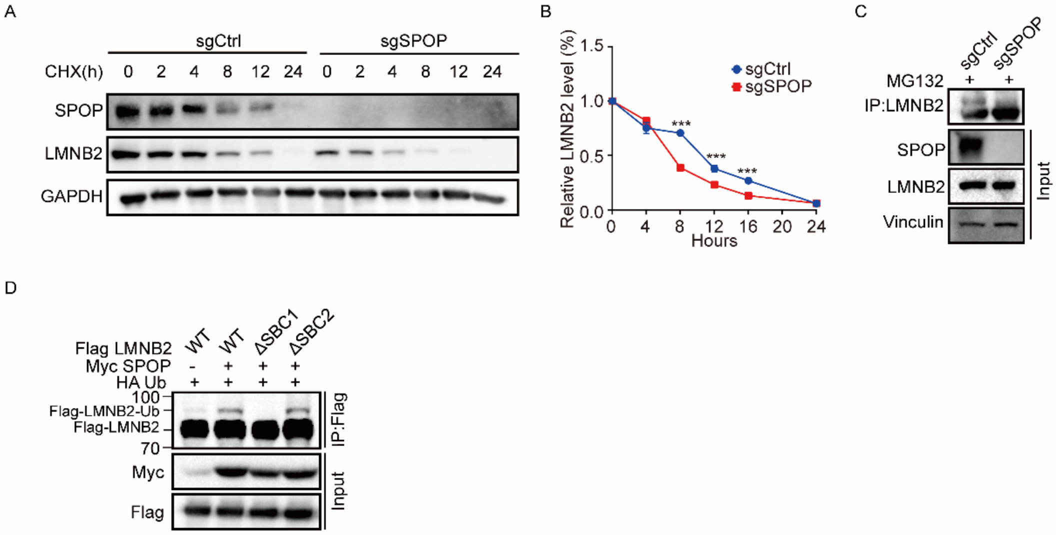

Supplemental Figure 4. SPOP maintains LMNB2 protein level by promoting its mono-ubiquitination at Lysine-484, Related to Figure 3. (**A** and **B**) IB analysis (**A**) and quantification (**B**) of KMNB2 protein in whole-cell lysates from 293T cells. Data were shown as the mean ± SD from three biological replicates ($n = 3$). (**C**) Co-IP analysis of 293T cells transfected with indicated constructs. (**D**) Co-IP analysis of 293T cells transfected with indicated constructs. (**B**) Statistical comparisons were performed using Two-way ANOVA followed by Tukey's multiple comparisons test. ***P < 0.001.

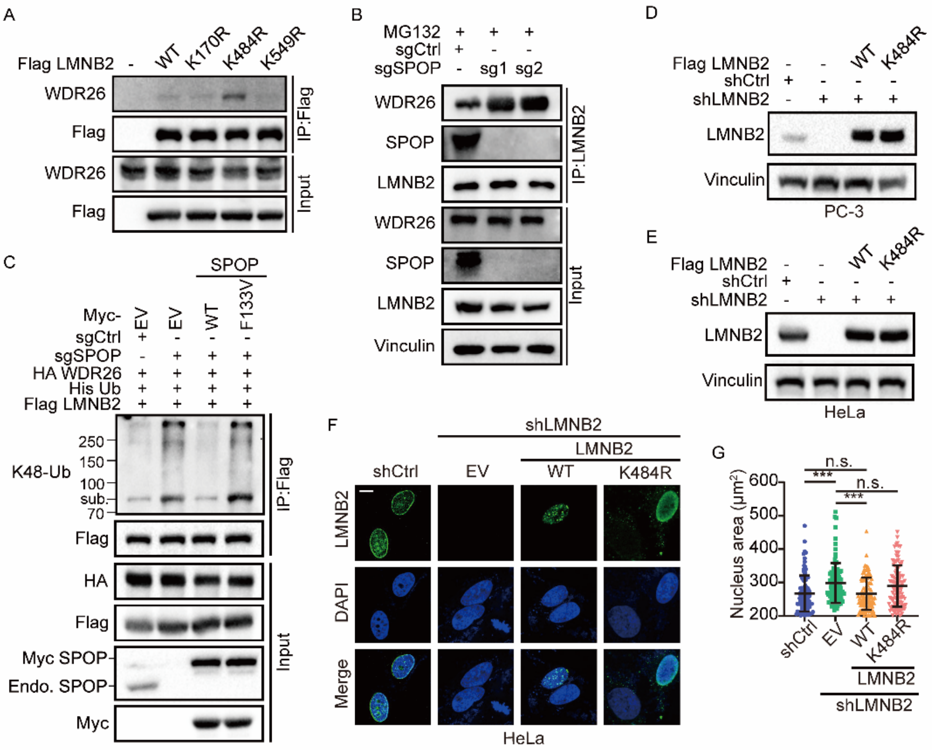

Supplemental Figure 5. Lys484 mono-ubiquitination stabilizes LMNB2 by antagonizing WDR26-mediated degradation, Related to Figure 4.
(**A**) Co-IP analysis of indicated proteins in 293T cells transfected with Flag-WT or mutated LMNB2 in combination with other constructs. (**B**) IB analysis of WCL derived from HeLa *control* and *SPOP* knockout cells. (**C**) Co-IP analysis of LMNB2 ubiquitination in 293T cells transfected with indicated constructs. (**D** and **E**) IB analysis of WCL derived from PC-3 (**D**) and HeLa (**E**) *control* and *SPOP* knockout cells transfected with indicated plasmids. (**F** and **G**) HeLa *control* and *LMNB2* knockdown cells transfected with Flag-WT or K484R LMNB2 were subjected to IF with representative images shown in (**F**) and quantification in (**G**). Scale bar, 10μm. Data were shown as the mean ± SD of three biological replicates ($n = 100$). (**G**) Statistical comparisons were performed using One-way ANOVA followed by Dunnett's multiple comparisons test. n.s., not significant, ***$P < 0.001$.

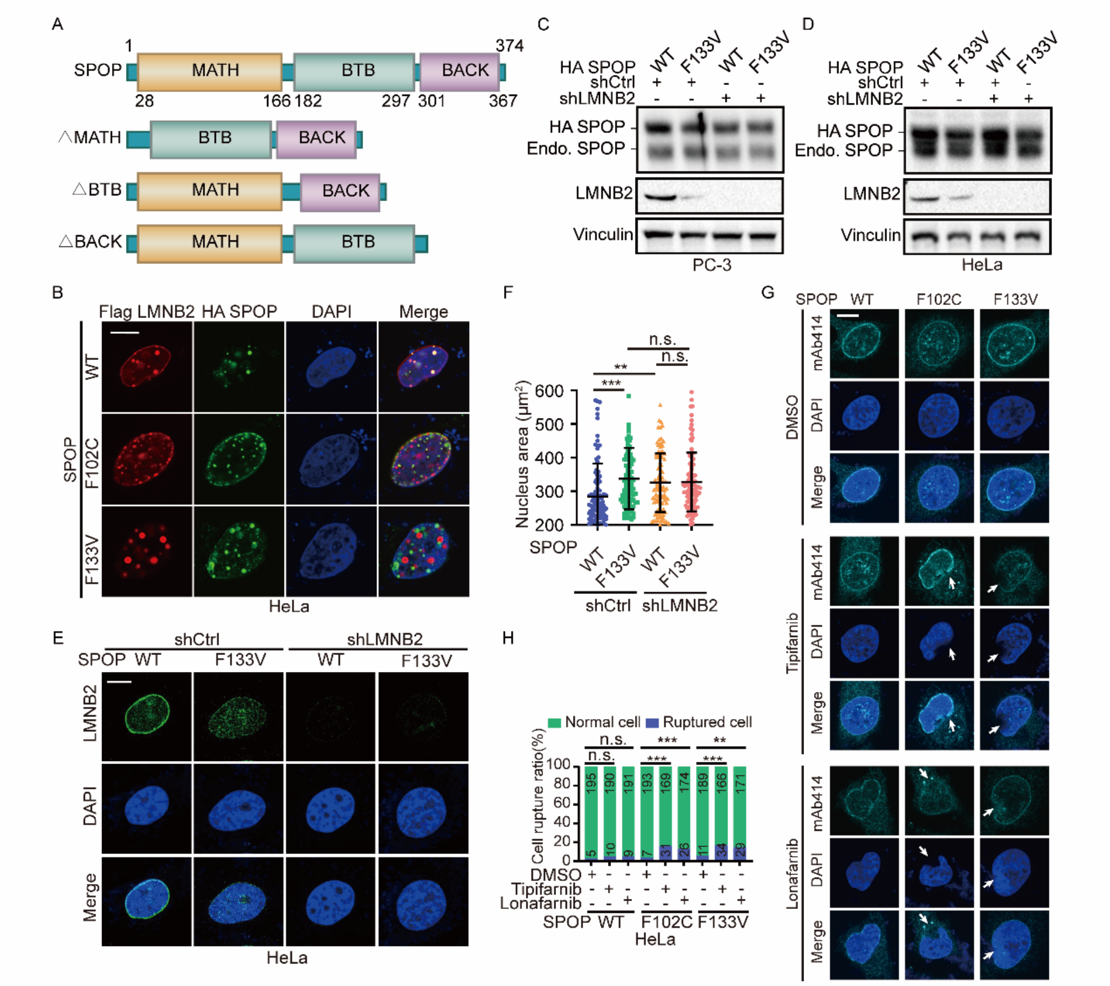

Supplemental Figure 6. SPOP mutation increases nuclear envelope rupture risk upon farnesyltransferase inhibition, Related to Figure 5. (**A**) Schematic diagram showing the full-length SPOP and the truncated SPOP constructs. (**B**) Representative images of IF of Flag LMNB2 and HA SPOP from HeLa cells. Scale bar, 10μm. (**C** and **D**) IB analysis of WCL derived from PC-3 (**C**) and HeLa (**D**) *control* and *LMNB2* knockdown cells infected with lentivirus expressing WT or F133V SPOP. (**E** and **F**) HeLa *control* and *LMNB2* knockdown cells infected with lentivirus expressing WT or F133V SPOP were subjected to LMNB2 IF with representative images shown in (**E**) and quantification in (**F**). Scale bar, 10μm. Data were shown as the mean ± SD of three biological replicates ($n = 100$). (**G** and **H**) PC-3 cells infected with lentivirus expressing HA-WT, F102C or F133V SPOP were subjected to mAb414 IF. DMSO, 10μM Tipifarnib or 5μM Lonafarnib was added for 24 hours before harvest. Representative images were shown in (**G**), quantification of the cell rupture ratio was shown in (**H**). Scale bar, 5μm. Data were shown as the mean ± SD of three biological replicates ($n = 200$). n.s., not significant, $^{**}P < 0.01$, $^{***}P < 0.001$ by One-way ANOVA followed by Dunnett’s multiple comparisons test (**F**) or Fisher's Exact Test(**H**).

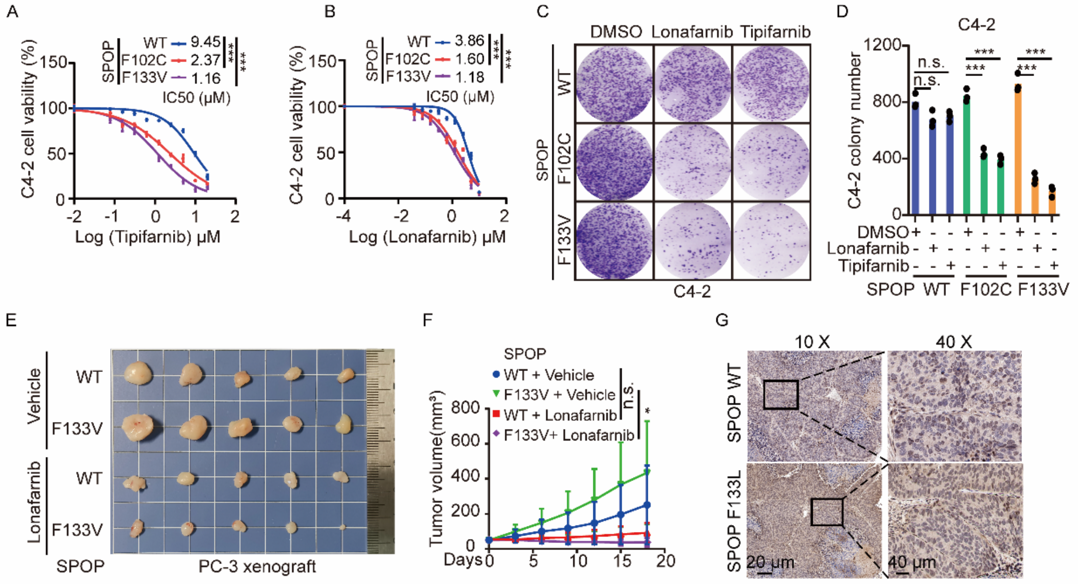

Supplemental Figure 7. SPOP mutant cells are hypersensitive to farnesyltransferase inhibition, Related to Figure 6.

(**A** and **B**) Dose-response survival curves of C4-2 cell lines infected with lentivirus expressing HA-WT, F102C or F133V SPOP exposed to increasing concentrations of Tipifarnib (**A**) or Lonafarnib (**B**). Data were shown as the mean ± SD of three independent experiments (*n* = 3). (**C** and **D**) Colony formation assays in C4-2 cell lines infected with lentivirus expressing HA-WT, F102C or F133V SPOP. The number of colonies was counted. Representative colonies are shown in (**C**), with quantification data shown in (**D**). Data were presented as the mean ± SD of three independent experiments (*n* = 3). (**E** and **F**) PC-3 SPOP WT or SPOP F133V Xenograft tumors were transplanted subcutaneously into SCID mice and treated with Lonafarnib (20 mg/kg, twice daily by oral gavage) or vehicle. Mice were treated for 18 days and then sacrificed. Tumors were isolated and are shown in (**E**). Volume of the tumors from (**E**) (*n* = 5) are shown in (**F**). All data are shown as mean ± SD. (**G**) AR staining of SPOP WT or SPOP F133L PDX tumor tissues. n.s., not significant, ***P < 0.001 by Two-way ANOVA (**A** and **B**) or One-way ANOVA followed by

Dunnett's multiple comparisons test (**D**). (**F**) Two-tailed unpaired Student's t-test. Comparing the size of tumors in different groups at day 18. *P < 0.05.

Supplemental Table 1. List of primers.

	Forward primers	Reverse primers
PCR		
LMNB2-K170R	CGGCGCGGCCTGGAGAGTGACGTG	GACGCGGCCTGGAGAGTGACGTG
LMNB2-K484R	CGGTTTGTGCAGCTCAAGAACAACT	GCCCTCCAGGTCGATCTC
LMNB2-K549R	AGAGGCCAGAGCAGCTGGGGCAC	CCACACCAGCGTCGAGG
LMNB2-TAA	GCTGCCAGCAGCGGCAAGCTTGTCCGC	GGTGGCTCGTGAGACGGTGACGCGCG
LMNB2-SBC1	CCGGCAGCTTGTCCGCCACCG	TCGTGAGACGGTGACGCGCG
LMNB2-SBC2	AGAGGCTGCTACGTGATG	GTCCCCCTGTTGGTGG
SPOP-F102C	TGTAAATTCTCCATCCTGAATGCCAAG	TTTTGCCCGAACTTCACTCTTTG
SPOP-F133V	GTGAAGAAATTCATCCGTAGAGATTTT	TCCCCAGTCTTTGCCTTGC
SPOP-Exon6	ACCCATAGCTTTGGTTTCTTCTCCC	TATCTGTTTTGGACAGGTGTTTGCG
SPOP-Exon7	ACTCATCAGATCTGGGAACTGC	AGTTGTGGCTTTGATCTGGTT
shRNA		
LMNB2 sh1	GCTCAAGAACAACTCGGACAA	TTGTCCGAGTTGTTCTTGAGC
LMNB2 sh2	GCAGCAGGAGTACGACTTCAA	TTGAAGTCGTACTCCTGCTGC
LMNB2 sh3	GGTCAACAAGAGCGCCAAGAA	TTCTTGGCGCTCTTGTTGACC
qRT-PCR		
LMNB2	GTCCTGGATGAGACGGCTC	GCGCTCTTGTTGACCTCGT
sgRNA		
SPOP sg1	CACCGCAAGCTTACCCTCTTCTGCG	AAACCGCAGAAGAGGGTAAGCTTGC
SPOP sg2	CACCGGTCATCAGGGAGAAGCCCGT	AAACACGGGCTTCTCCCTGATGACC

Supplemental Table 2. List of SPOP wild-type and mutant prostate cancer patient samples

Patient ID	SPOP Mutation	Patient ID	SPOP Mutation	Patient ID	SPOP Mutation
1	F102C	35	N/A	69	N/A
2	N/A	36	N/A	70	N/A
3	N/A	37	N/A	71	N/A
4	N/A	38	F102C	72	N/A
5	N/A	39	N/A	73	N/A
6	F133L	40	F102C	74	N/A
7	F102C	41	N/A	75	N/A
8	N/A	42	N/A	76	N/A
9	N/A	43	N/A	77	N/A
10	N/A	44	N/A	78	F102C
11	N/A	45	N/A	79	N/A
12	N/A	46	N/A	80	N/A
13	W131*	47	N/A	81	N/A
14	N/A	48	N/A	82	N/A
15	F102C	49	N/A	83	N/A
16	N/A	50	N/A	84	N/A
17	N/A	51	F102C	85	N/A
18	F102C	52	F102C	86	N/A
19	N/A	53	N/A	87	N/A
20	F102C	54	N/A	88	N/A
21	N/A	55	N/A	89	W131C
22	N/A	56	N/A	90	N/A
23	F102C	57	N/A	91	N/A
24	N/A	58	N/A	92	N/A
25	F133L	59	N/A	93	N/A
26	F102C	60	N/A	94	N/A
27	F102C	61	N/A	95	N/A
28	N/A	62	N/A	96	N/A
29	N/A	63	N/A	97	N/A
30	W131G	64	N/A	98	N/A
31	N/A	65	N/A	99	N/A
32	F102C	66	N/A	100	N/A
33	N/A	67	N/A		
34	F102C	68	N/A		

*Termination codon
