# Supplementary material for: Cancer-associated SPOP mutations enlarge nuclear size and facilitate nuclear envelope rupture upon farnesyltransferase inhibitor treatment
Source: J Clin Invest. 2025 Jul 15;135(14):e189048. doi: 10.1172/JCI189048 (PMC12259254; doi:10.1172/JCI189048)

Unedited blot and gel images

Fig.1

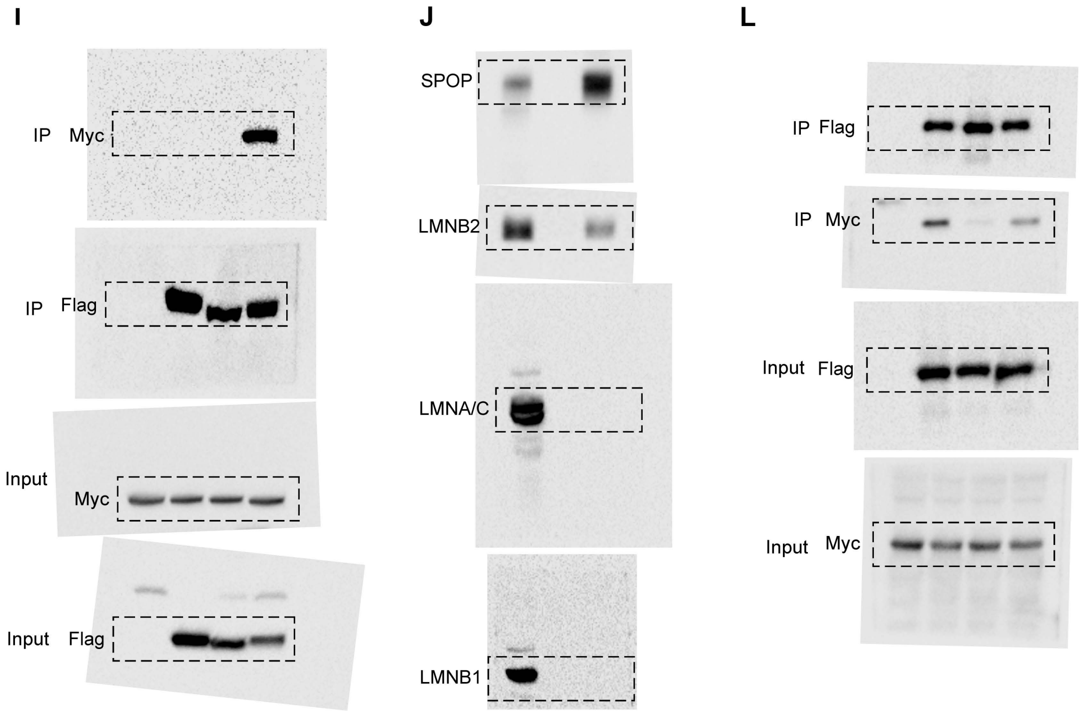
I
IP Myc
IP Flag
Input Myc
Input Flag
J
SPOP
LMNB2
LMNA/C
LMNB1
L
IP Flag
IP Myc
Input Flag
Input Myc

Fig.2

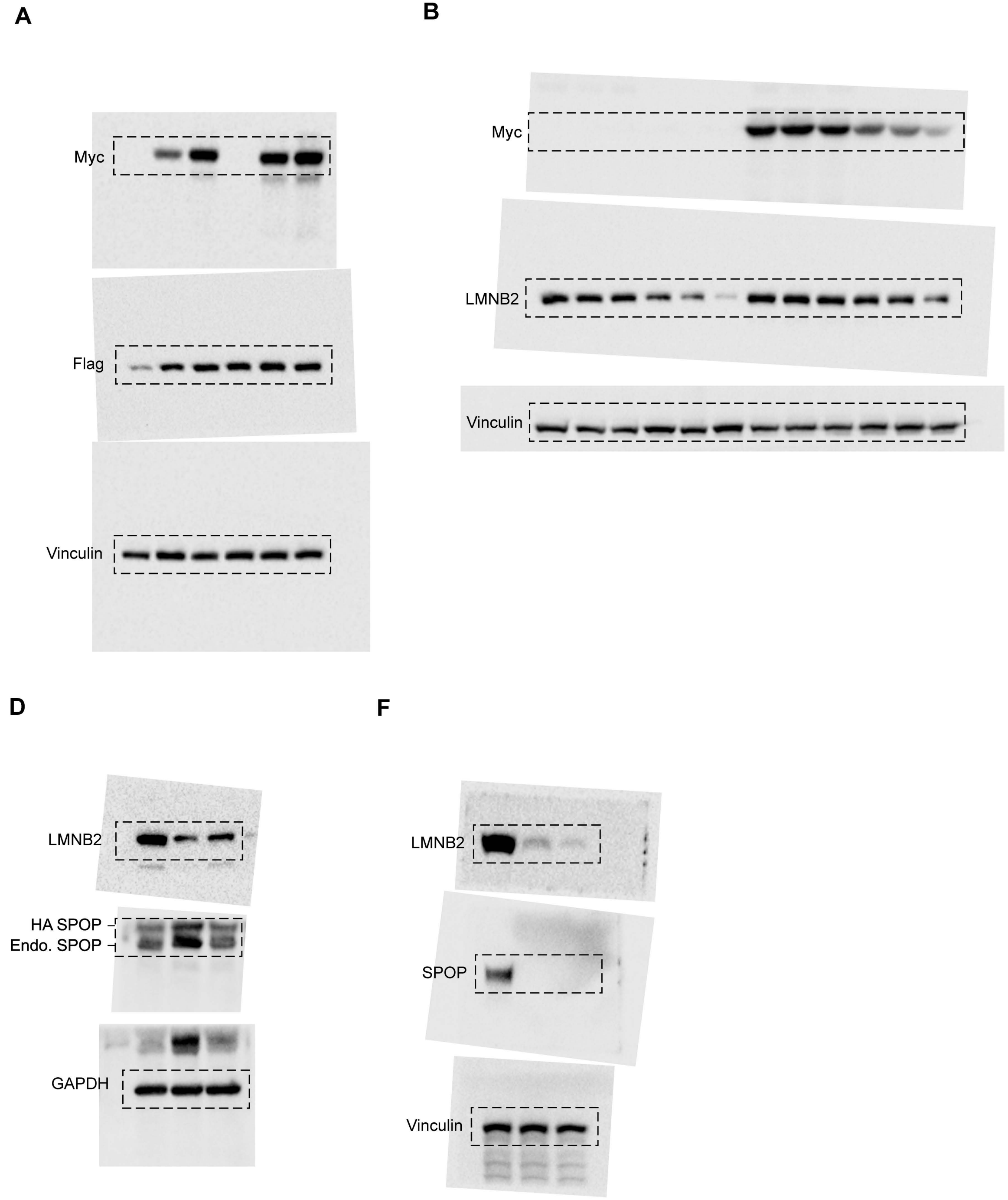
A
Myc
Flag
Vinculin
B
Myc
LMNB2
Vinculin
D
LMNB2
HA SPOP
Endo. SPOP
GAPDH
F
LMNB2
SPOP
Vinculin

Fig.3

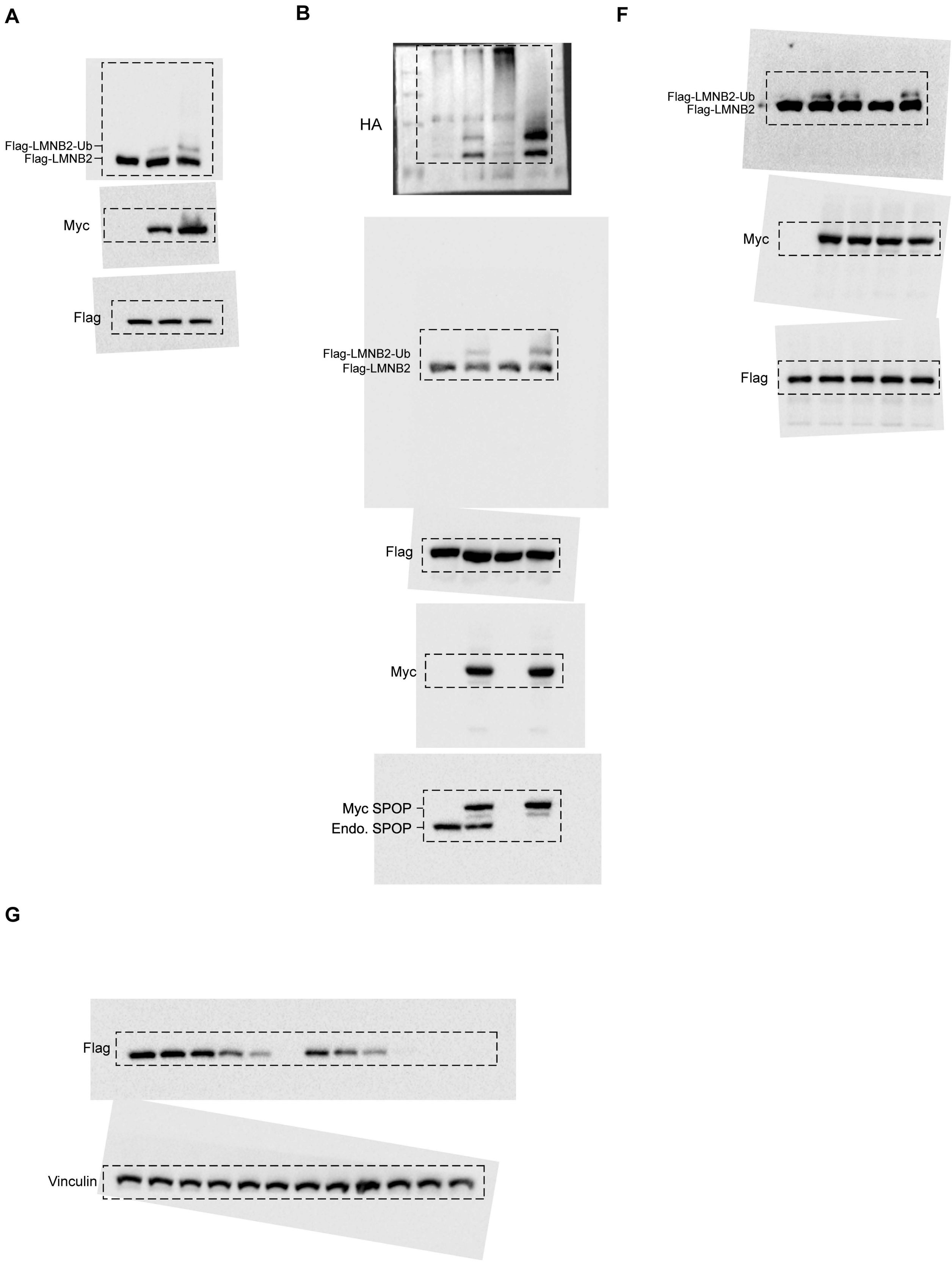
A
Flag-LMNB2-Ub
Flag-LMNB2
Myc
Flag
B
HA
Flag-LMNB2-Ub
Flag-LMNB2
Flag
Myc
Myc SPOP
Endo. SPOP
F
Flag-LMNB2-Ub
Flag-LMNB2
Myc
Flag
G
Flag
Vinculin

Fig.4

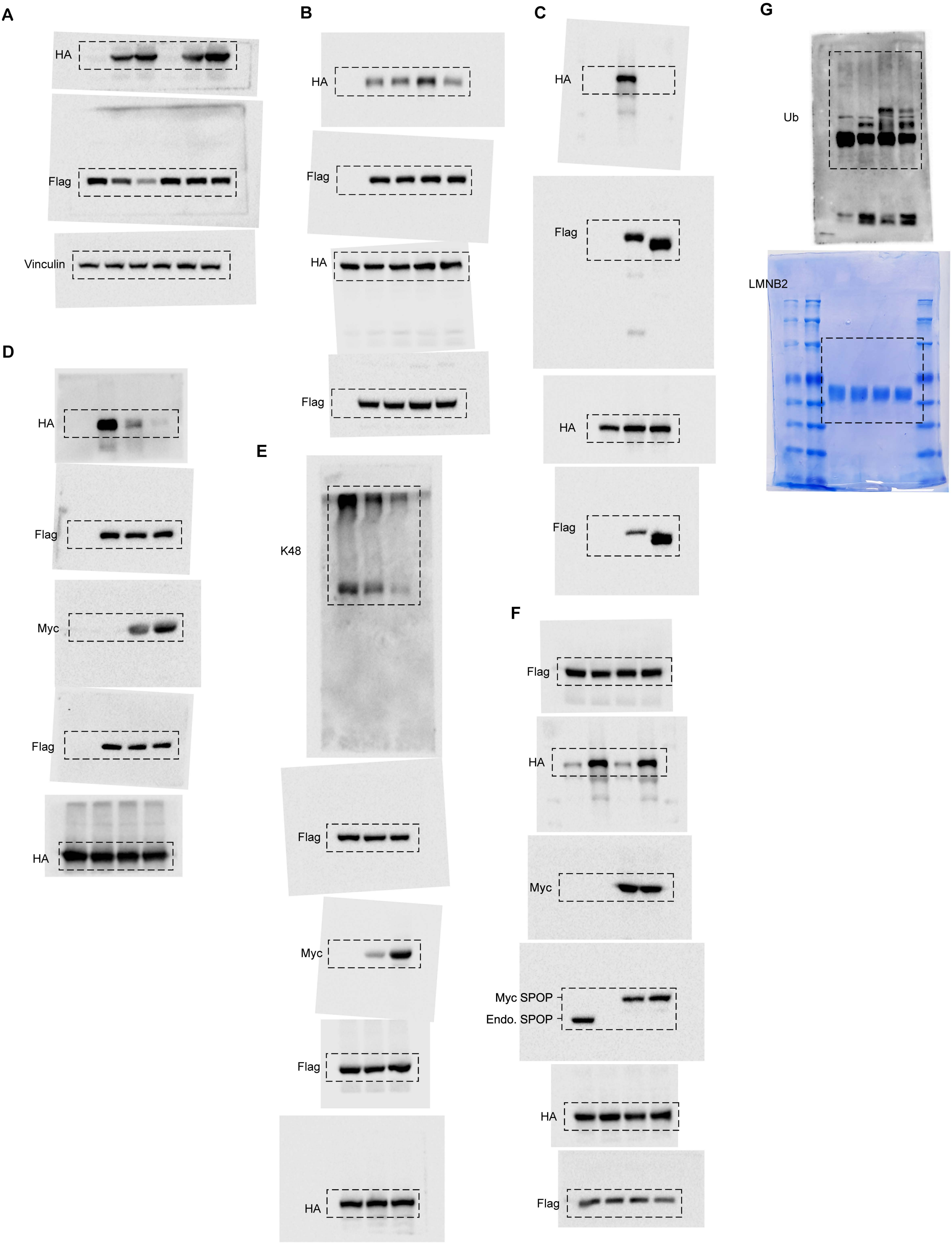
A
HA
Flag
Vinculin
B
HA
Flag
HA
Flag
C
HA
Flag
HA
Flag
D
HA
Flag
Myc
Flag
HA
E
K48
Flag
Myc
Flag
HA
F
Flag
HA
Myc
Myc SPOP
Endo. SPOP
HA
Flag
G
Ub
LMNB2

Fig.5

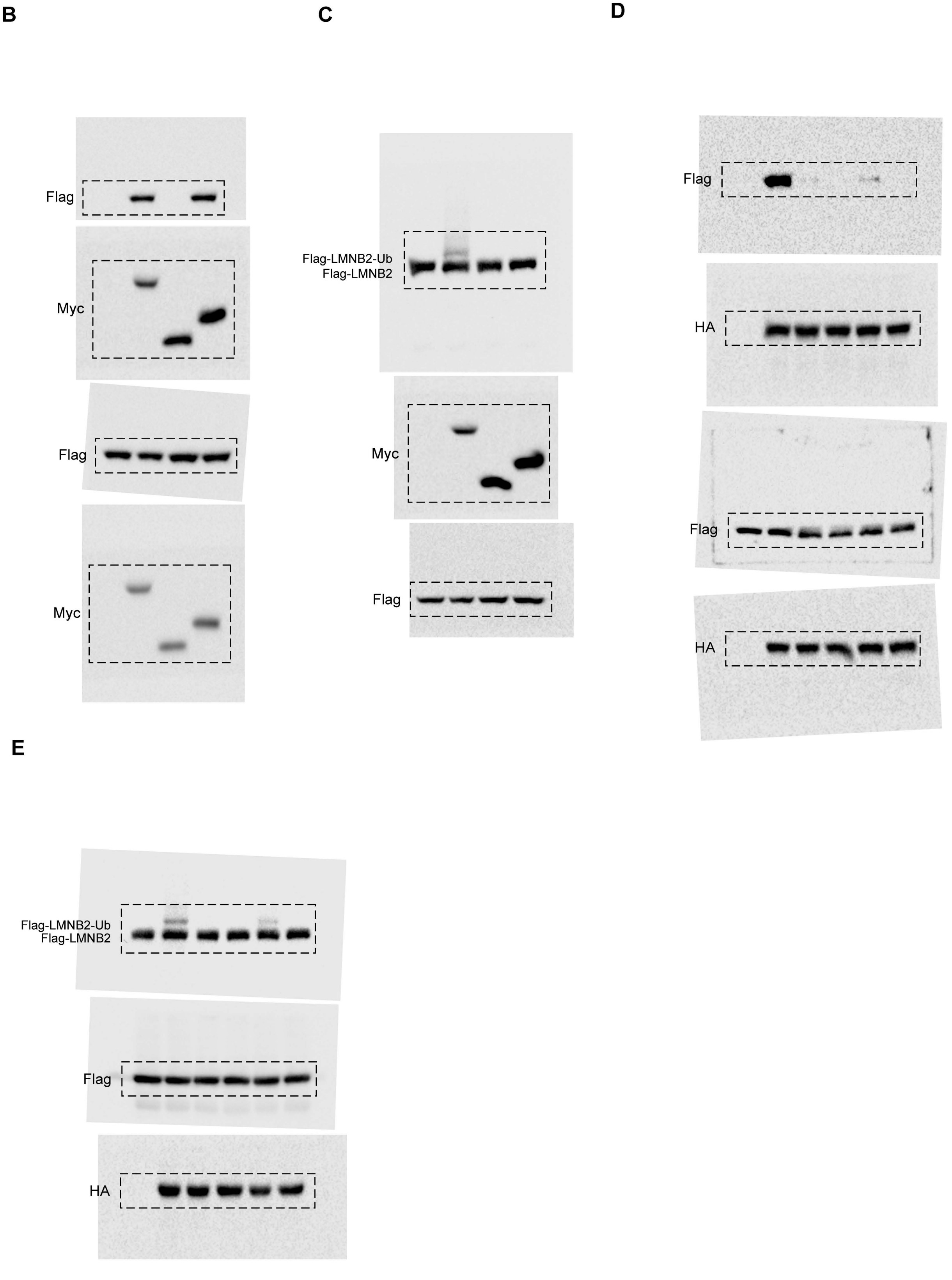
B
Flag
Myc
Flag
Myc
C
Flag-LMNB2-Ub
Flag-LMNB2
Myc
Flag
D
Flag
HA
Flag
HA
E
Flag-LMNB2-Ub
Flag-LMNB2
Flag
HA

Fig.S1

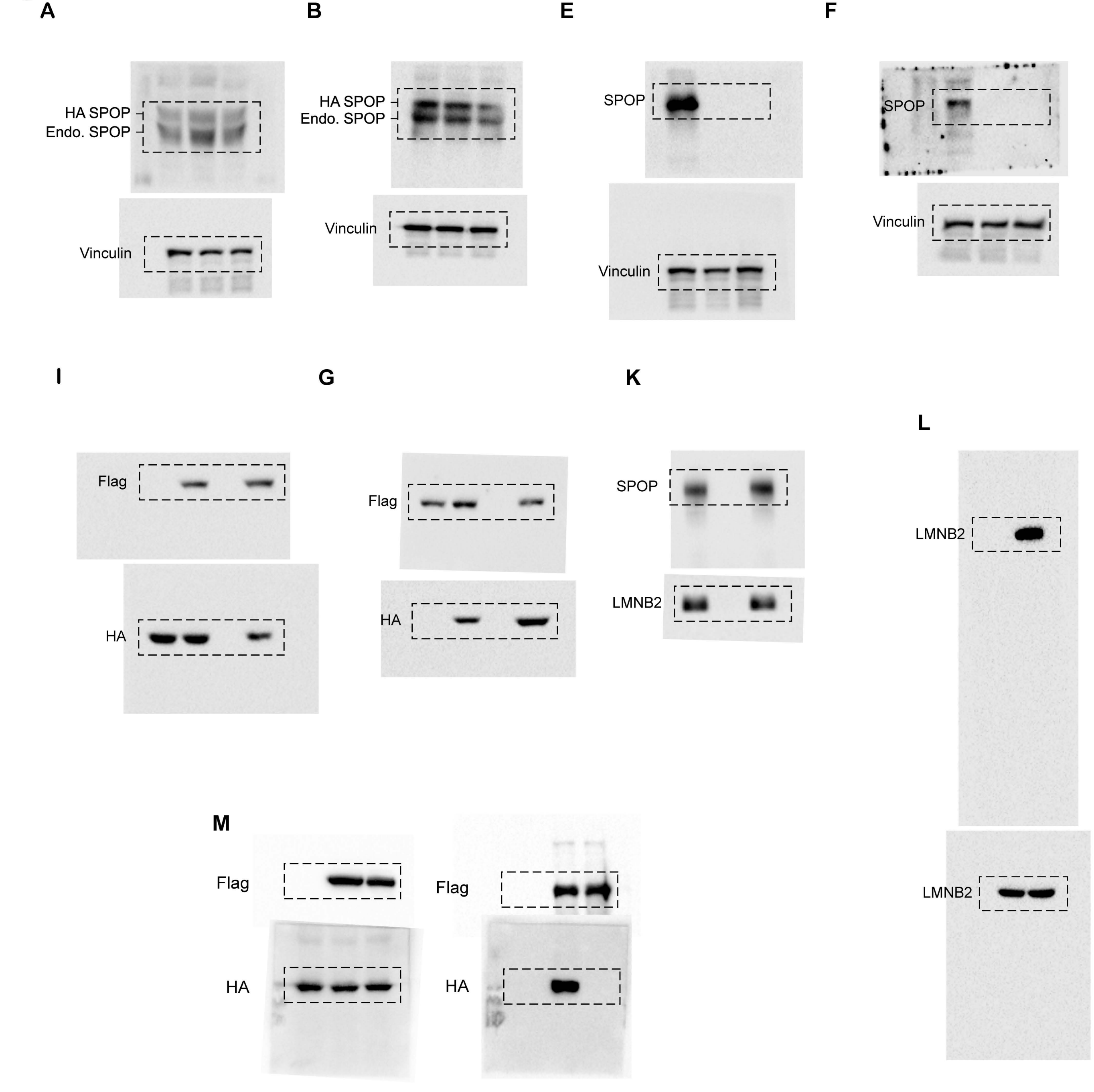
A
HA SPOP
Endo. SPOP
Vinculin
B
HA SPOP
Endo. SPOP
Vinculin
E
SPOP
Vinculin
F
SPOP
Vinculin
I
Flag
HA
G
Flag
HA
K
SPOP
LMNB2
L
LMNB2
LMNB2
M
Flag
HA
Flag
HA

Fig. S2

S2A

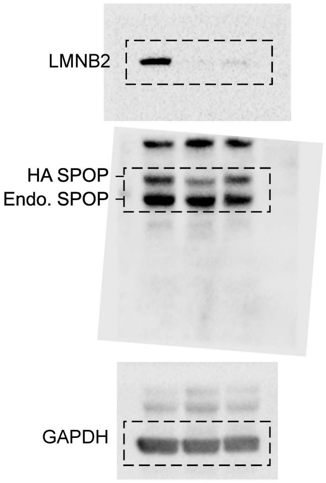

S2C

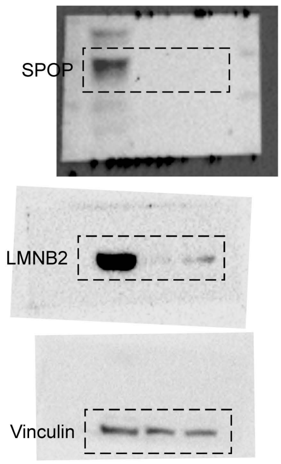

S2E

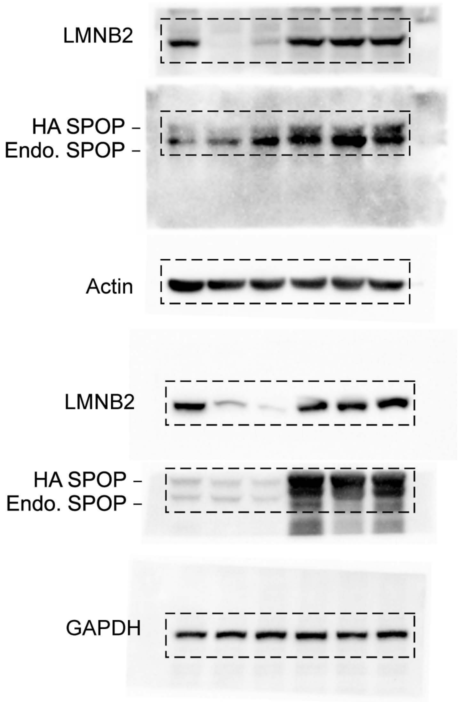

S2F

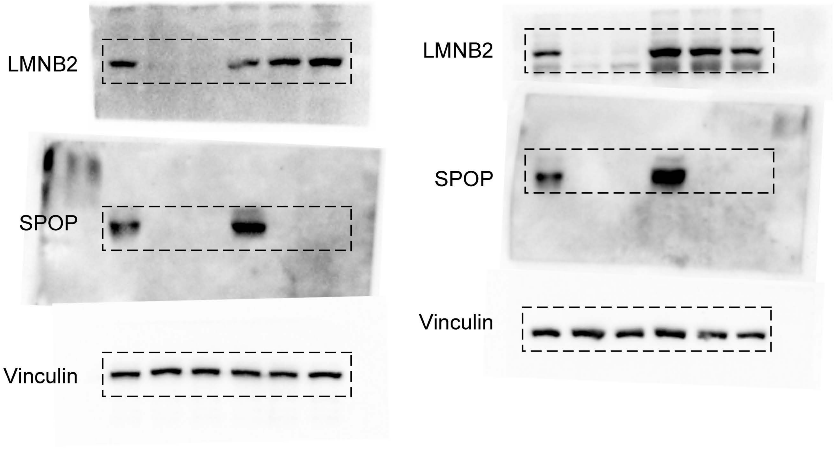

Fig. S3

S3A

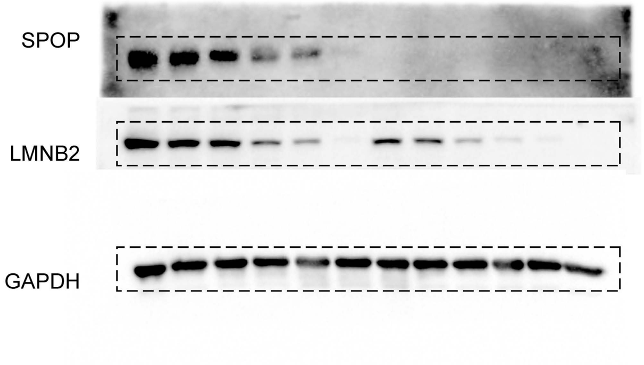

S3C

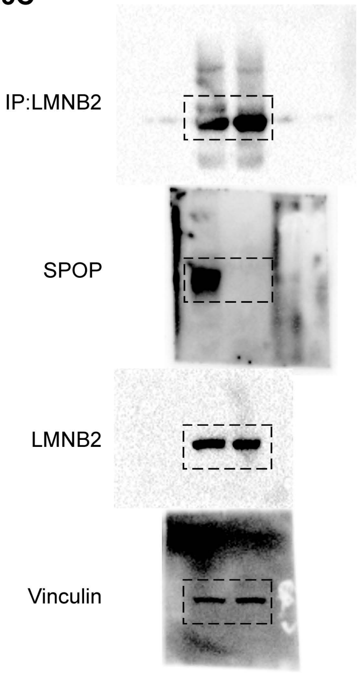

S3D

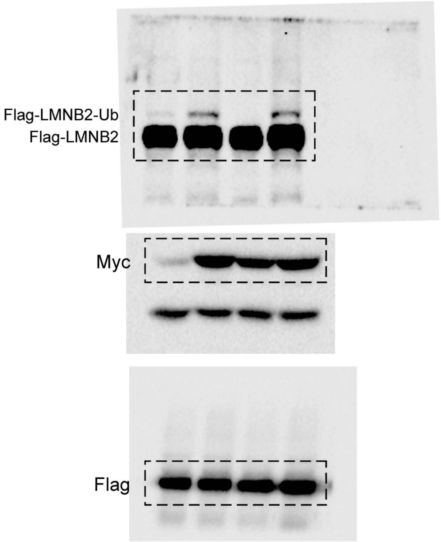

Fig. S4

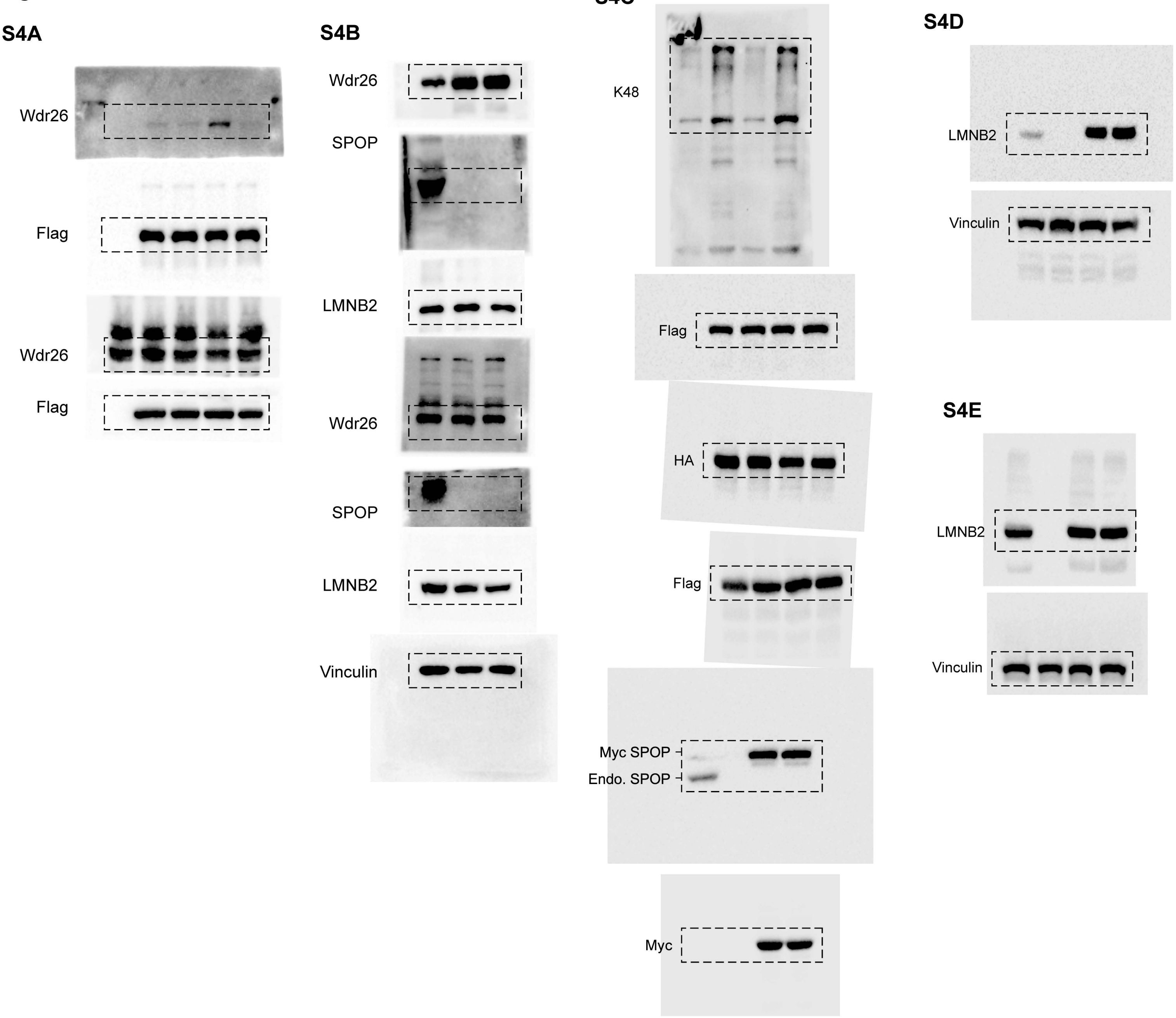
S4A
Wdr26
Flag
Wdr26
Flag
S4B
Wdr26
SPOP
LMNB2
Wdr26
SPOP
LMNB2
Vinculin
S4C
K48
Flag
HA
Flag
Myc SPOP
Endo. SPOP
Myc
S4D
LMNB2
Vinculin
S4E
LMNB2
Vinculin

Fig. S5

S5C

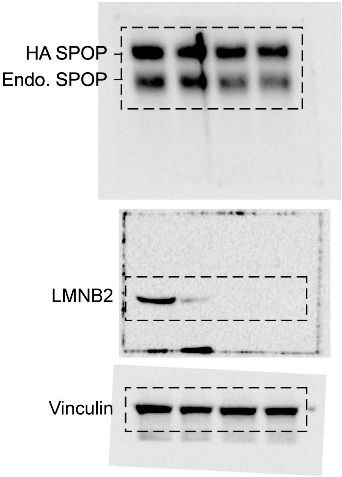

S5D

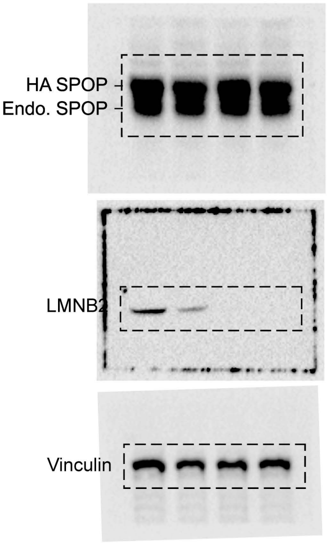

Supplement: Unedited blot and gel images [file jci-135-189048-s119.pdf]
